# Role of the dynamic tumor microenvironment in controversies regarding immune checkpoint inhibitors for the treatment of non-small cell lung cancer (NSCLC) with EGFR mutations

**DOI:** 10.1186/s12943-019-1062-7

**Published:** 2019-09-16

**Authors:** Anqi Lin, Ting Wei, Hui Meng, Peng Luo, Jian Zhang

**Affiliations:** Department of Oncology, Zhujiang Hospital, Southern Medical University, 253 Industrial Avenue, Guangzhou, 510282 Guangdong China

**Keywords:** Tumor microenvironment, EGFR mutations, Non-small cell lung cancer, Immunotherapy, Anti-PD-1/PD-L1 treatment

## Abstract

Immunotherapy has been incorporated into the first- and second-line treatment strategies for non-small cell lung cancer (NSCLC), profoundly ushering in a new treatment landscape. However, both adaptive signaling and oncogenic (epidermal growth factor receptor (EGFR)-driven) signaling may induce PD-L1 upregulation in NSCLC. Nevertheless, the superiority of immune checkpoint inhibitors (ICIs) in advanced EGFR-mutant NSCLC is only moderate. ICIs appear to be well tolerated, but clinical activity for some advanced EGFR-mutant NSCLC patients has only been observed in a small proportion of trials. Hence, there are still several open questions about PD-L1 axis inhibitors in patients with NSCLC whose tumors harbor EGFR mutations, such as the effect of EGFR tyrosine kinase inhibitors (TKIs) or EGFR mutations in the tumor microenvironment (TME). Finding the answers to these questions requires ongoing trials and preclinical studies to identify the mechanisms explaining this possible increased susceptibility and to identify prognostic molecular and clinical markers that may predict benefits with PD-1 axis inhibition in this specific NSCLC subpopulation. The presence of multiple mechanisms, including dynamic immune TME profiles, changes in PD-L1 expression and low tumor mutational burdens, may explain the conflicting data regarding the correlation between PD-L1 axis inhibitors and EGFR mutation status. We conducted a review of this currently controversial topic in an attempt to aid in the decision-making process.

## Introduction

Lung cancer is the most common malignant tumor (11.6%) and the leading cause of cancer-related death (18.4%) worldwide [1]. According to the latest International Agency for Research on Cancer (IARC) report, there are approximately 2.1 million lung cancer patients worldwide. In 2018, there were an estimated 2,093,876 new cases of lung cancer worldwide and approximately 1,761,007 deaths [1]. Eighty percent of new lung cancer patients are diagnosed with NSCLC [2], which does not have obvious clinical results and/or symptoms in the early stage. When patients are diagnosed with NSCLC, the optimal treatment period is often missed. Seventy-five percent of NSCLC is diagnosed at an advanced stage, resulting in a 5-year survival rate of less than 15% [3, 4].

Recent advances in next-generation sequencing (NGS), other high-throughput genomic profiling platforms and the generation of multiple genetically engineered mouse models (GEMMs) of lung cancer have allowed researchers to transform the view of NSCLC from histopathological descriptions to precise molecular and genetic identities that can be resolved at the single-cell level [5]. Following the identification of KRAS and BRAF mutations, EGFR mutations were discovered in patients with lung adenocarcinoma (ADC) and were associated with the response to EGFR inhibitors. Given this relatively large number of mutations per tumor, the treatment of NSCLC has entered a new revolutionized era of molecular targeted therapy [5]. Currently, EGFR tyrosine kinase inhibitors (EGFR-TKIs) are recommended by clinical guidelines as first-line therapeutic drugs [6–8] for advanced NSCLC patients with EGFR-sensitive mutations and no resistance genes. Compared to chemotherapy, EGFR-TKIs have demonstrated superior survival [9] in terms of the objective response rate (ORR) (67.0% vs 40.8%) and median progression-free survival (PFS) (10.9 months vs 7.4 months). However, these compounds have provided only initial improvement in clinical outcomes, and acquired resistance within 9–14 months is almost inevitable [10–12]. An innovative treatment for overcoming EGFR-TKI resistance remains an unresolved issue. This topic has gained increasing attention for strengthening the potential benefits of immunotherapy [13]. ICIs have already shown excellent survival benefits for NSCLC patients with long-term efficacy and less toxicity [14–22]. For example, the longest follow-up analysis of data from a clinical trial showed that 129 patients with advanced NSCLC who had failed multiple treatments had a 5-year survival rate of 26% after receiving nivolumab [16], which was much higher than the 5-year survival rate of 1–8% in NSCLC patients who did not receive ICIs [23, 24]. Furthermore, preclinical results have shown that EGFR activation can upregulate intrinsic PD-L1 expression on tumor cells, which induces T cell apoptosis and contributes to the immune escape of EGFR-mutant NSCLC. In addition, EGFR-TKIs can potentiate the induction of MHC class I and II molecules in response to IFN-γ and enhance T cell-mediated tumor killing [47]. In this regard, these studies provide a theoretical basis to support the potential synergistic effects of combining PD-1/PD-L1 inhibitors and EGFR-targeted therapy in NSCLC patients carrying EGFR mutations accompanied by upregulation of PD-L1 expression.

A number of related studies to evaluate the safety and efficacy of immunotherapy combined with targeted therapy in patients with EGFR mutations are currently underway. Most recent clinical trials have shown that patients with EGFR mutations are unable to benefit from immunotherapy. Intriguingly, immunotherapy in these patients may be associated with the development of hyperprogressive disease (HPD) and lead to increased toxic effects [25, 26]. The CAURAL trial is a multiphase III trial in which osimertinib is combined with durvalumab. Both EGFR-TKI-sensitizing- and EGFR T790 M mutation-positive advanced patients were included in the study, though the results did not show a benefit for the combination arms with regard to ORR (64% vs 80%), duration of response (DOR) (17.5 months vs 21.4 months) or disease control rate (DCR) (93% vs 100%), which was even lower than that of the osimertinib monotherapy treatment group [27]. In addition, the KEYNOTE-021 study [28] was conducted to test the efficacy of combination pembrolizumab with erlotinib in EGFR-mutant advanced NSCLC patients. The combination treatment enhanced the median PFS (19.5 months) benefit along with ORR (41.7%) compared with that of patients taking first-generation EGFR-TKIs (11.0 months) or osimertinib (19.2 months) [29]. Moreover, preliminary results from other early studies have shown promising efficacy and acceptable toxicity. Specifically, in the phase I study of nivolumab (CheckMate 012), 21 EGFR-mutant NSCLC patients were treated with the combination of nivolumab and erlotinib associated with an acceptable toxicity profile, with a 15% ORR, 65% DCR, 5.1-month median PFS and 18.7-month median overall survival (OS) [30]. This trial also reported one TKI-naive patient who was effectively treated with nivolumab plus erlotinib, with an ongoing response lasting more than 5 years. However, others have demonstrated the opposite result (Table 1). Overall, the combined use of PD-1/PD-L1 inhibitors and EGFR-TKIs remains controversial.

**Table 1:** Summary of Completed or Ongoing Clinical Trials of Immune Checkpoint Inhibitors in Combination with or without EGFR-TKIs in Locally Advanced or Metastatic NSCLC

Clinical Trial	Number of patients	Median age (yr)	Male Sex (%)	Baseline	Treatment	ORR (%)	Median OS (months)	Median PFS (months)	Phase	Status
NCT02088112 [188]	10	NA	NA	TKI-naive EGFR (+)	Concurrent Gefitinib+Durvalumab	77.8	NA	NA	1	Active:not recruiting
	10	NA	NA	TKI-naive EGFR (+)	Priming Gefitinib monotherapy followed by concurrent Durvalumab+Gefitinib	90	NA	NA	1	Active:not recruiting
NCT02040064/GEFTREM [189]	18	66	35	TKI-pretreated EGFR (+)	Gefitinib+ Tremelimumab	50–80	NA	NA	1	Completed
NCT02574078/CheckMate 370 [190]	NA	NA	NA	TKI-naive EGFR (+)	Nivolumab + Erlotinib	NA	NA	NA	1/2	Active:notrecruiting
	13	63	54	TKI-naive EGFR (+)	Nivolumab + Crizotinib	NA	NA	NA	1/2	Active:notrecruiting
	NA	NA	NA	TKI-naive EGFR (+)	Nivolumab	NA	NA	NA	1/2	Active:notrecruiting
NCT01454102/CheckMate 012 [191]	21	NA	NA	TKI-naive EGFR (+)	Nivolumab + Erlotinib	19	NA	NA^a^	1	Active:not recruiting
NCT02013219 [192]	28	61	NA	TKI-naive EGFR (+) and treatment-naive ALK (+)	Atezolizumab+Erlotinib	75	NA	11.3	1	Active:not recruiting
NCT02039674/KEYNOTE-021 [27]	12	60	50	TKI-naive EGFR (+)	Pembrolizumab+Erlotinib	41.7	2	19.5	1/2	Active:not recruiting
	7	68	43	TKI-naive EGFR (+)	Pembrolizumab+Gefitinib	14.3	3	1.4	1/2	Active:not recruiting
NCT02143466/TATTON [193]	10	67	30	TKI-pretreated EGFR (+)	Osimertinib+Durvalumab	39–70	NA	NA	1	Active:not recruiting
	13	58	46	TKI-pretreated EGFR (+)					1	Active:not recruiting
	11	57	55	TKI-naive EGFR (+)					1	Active:not recruiting
NCT02454933/CAUREL [27]	17	65	24	TKI-pretreated EGFR (T790 M+)	Osimertinib	80	NA	NA	3	Active:not recruiting
	12	56	50		Osimertinib+Durvalumab	64	NA	NA	3	Active:not recruiting
NCT01642004/CheckMate-017 [15]	135	62	82	Platinum-Based Chemotherapy Pretreated	Nivolumab	20	9.2	3.5	3	Active:not recruiting
	137	64	71		Docetaxel	9	6	2.8	3	Active:not recruiting
NCT01673867/CheckMate-057 [16]	292	61	52	Platinum-Based Chemotherapy Pretreated	Nivolumab	19	12.2	2.3	3	Active:not recruiting
	290	64	58		Docetaxel	12	10.4	4.2	3	Active:not recruiting
NCT01905657/Keynote-010 [17]	345	63	62	Platinum-Based Chemotherapy Pretreated	Pembrolizumab	9–18	8.5–12.7	4	2/3	Active:not recruiting
	346	63	62						2/3	Active:not recruiting
	343	62	61		Docetaxel	18	10.4	3.9	2/3	Active:not recruiting
NCT02008227/OAK [18]	425	63	61	Platinum-Based Chemotherapy Pretreated	Atezolizumab	14	13.8	2.8	3	Completed
	425	64	61		Docetaxel	13	9.6	4	3	Completed
NCT01903993 /POPLAR [19]	144	62	65	Platinum-Based Chemotherapy Pretreated	Atezolizumab	14	12.6	2.7	2	Completed
	143	62	53		Docetaxel	13	9.7	3	2	Completed
NCT02087423/ATLANTIC [194]	111	61	NA	Pretreated-EGFR (+)/ALK(+)	Durvalumab	NA	13.3	NA	2	Active:not recruiting
	265	NA	NA	Pretreated-EGFR (−)/ALK(−)	Durvalumab	NA	NA	NA	2	Active:not recruiting
	68	61	NA	Pretreated- EGFR (−)/ALK(−)^b^	Durvalumab	NA	13.2	NA	2	Active:not recruiting
NCT01295827/Keynote-001 [180]	4	NA	NA	TKI-naive EGFR (+)	Pembrolizumab	50	18.6	5.3	1	Completed
	26	NA	NA	TKI-pretreated EGFR (+)	Pembrolizumab	4	4	1.9	1	Completed
NCT02366143/IMpower150 [187]	45	63	38	Chemotherapy-naiveEGFR (+)	Atezolizumab+Carboplatin+ Paclitaxel	36	21.4	6.9	2	Active, not recruiting
	34	64	18		Atezolizumab + Carboplatin + Paclitaxel + Bevacizumab	71	NE	10.2	2	
	45	61	21		Carboplatin+ Paclitaxel + Bevacizumab	42	18.7	6.9	2	
NCT02367781/IMpower130 [195]	32	NA	NA	EGFR (+)/ALK(+)	Atezolizumab + Carboplatin + Paclitaxel	NA	14.4	7.0	2	Active, not recruiting
	12	NA	NA		Carboplatin + Paclitaxel	NA	10.0	6.0	2	

*EGFR* Epidermal growth factor receptor, *OS* Overall survival, *PFS* Progression-free survival, *ORR* Objective Response Rate, *ALK* Anaplasticlymphoma kinase, *EGFR-TKI* Epidermal growth factor receptor-tyrosine kinase inhibitor, *NA* Not avaliable, *NR* Not reached, *NE* Not estimable, *NSCLC* Non-small cell lung cancer, *PD-L1* Programmed death-ligand 1

^a^24-week PFS rate was 51%

^b^received≥2 prior systemic treatment regimens+ ≥ 90% of TC expressing PD-L1

Recent preclinical and clinical studies have begun to reveal limited benefit of immune checkpoint inhibitors in EGFR-mutant NSCLC patients. Several reports have reported that the tumor microenvironment (TME) [31–36], tumor immunogenicity [37–39], tumor-specific mutations, copy number variants [40, 41] and abundances of specific intestinal bacteria can [42] affect the efficacy of ICIs. Multiple studies have demonstrated that EGFR mutations in NSCLC are more likely to correlate with an immunosuppressive TME [41, 43–48], the tumor mutation burden (TMB) [43, 49], and expression of PD-L1 [41, 50–53]. In addition, EGFR-TKIs may modulate the immune response by regulating TME. These factors are continuous variables in space and time, and the exact boundaries and correlations among them are still unclear [54]. The above findings might explain the contradictory clinical results for ICIs combined with EGFR-TKIs among patients with newly diagnosed or treated EGFR-mutant NSCLC.

In this review, we endeavor to compare and analyze all preclinical and clinical studies on the feasibility of treatment with ICIs or combined with EGFR-TKIs in NSCLC patients with EGFR mutations. We explore the unique TME of these patients, which may cause an inferior response to ICIs. We critically discuss the mechanisms underlying contradictory results in monotherapy and combination therapy and focus on improving the effectiveness of immunotherapy in EGFR-mutant NSCLC. Additional studies are warranted to further discover and identify prognostic biomarkers in patients with advanced EGFR-mutant NSCLC and to predict the benefits of anti-PD-1/PD-L1 treatment in this special NSCLC subpopulation.

## EGFR mutations affect the efficacy of anti-PD-1/PD-L1 treatment

Several studies have demonstrated the possible poor efficacy of PD-1 inhibitors for treating EGFR-mutant NSCLC patients [14, 40, 51]. Two meta-analyses on the efficacy of ICIs versus docetaxel in patients with pre-treated advanced NSCLC have been recently reported [39, 50]. In a report by Lee [50], there was a 32% reduction in the risk of death with ICIs compared with docetaxel in the intention to treat population (HR = 0.68, 95% CI: 0.61–0.77, *P <* 0.0001). Checkpoint inhibitors prolonged OS in the wildtype EGFR subgroup (HR = 0.66, 95% CI: 0.58–0.76, *P <* 0.0001) but not in the mutant EGFR subgroup (HR = 1.05, 95% CI: 0.70–1.55, *P =* 0.81). A similar analysis confirmed that ICIs do not enhance OS in NSCLC patients with EGFR mutations compared with that in patients taking docetaxel (HR = 1.09, 95% CI: 0.84–1.41) [39]. Another meta-analysis covering five clinical trials (Checkmate 017 and 057, Keynote 010, OAK, POPLAR) [14] also verified that patients with EGFR-sensitive mutations dramatically responded to docetaxel compared with PD-1 inhibitors (HR = 0.69, 95% CI:0.63–0.75; *P <* 0.001). Thus, a key question remains as to whether the benefit of ICIs among NSCLC patients with EGFR mutations is limited.

## Underlying mechanisms for the poor efficacy of anti-PD-1/PD-L1 treatment in EGFR-mutant NSCLC

### EGFR mutations affect the TME in NSCLC

The TME is the internal environment in which tumor cells depend on survival and development. TME is critical for the development of tumor immunotherapy strategies, and T lymphocytes, myeloid cells, cytokines, and exosomes constitute the immune regulatory networks [55, 56] of the TME. With tumor development and the plasticity of immune cells, T lymphocytes switch from having immune surveillance to immune escape [57, 58] functions via immunoediting and even exhibit immunosuppressive functions such as inducing regulatory T (Tregs) cells and upregulating myeloid-derived suppressor cells (MDSCs) [57, 59–63]. In addition, inflammatory cells and immunomodulatory mediators in the TME may be involved in an important mechanism to mediate tumor progression [60, 61, 64].

**Fig. 1:**
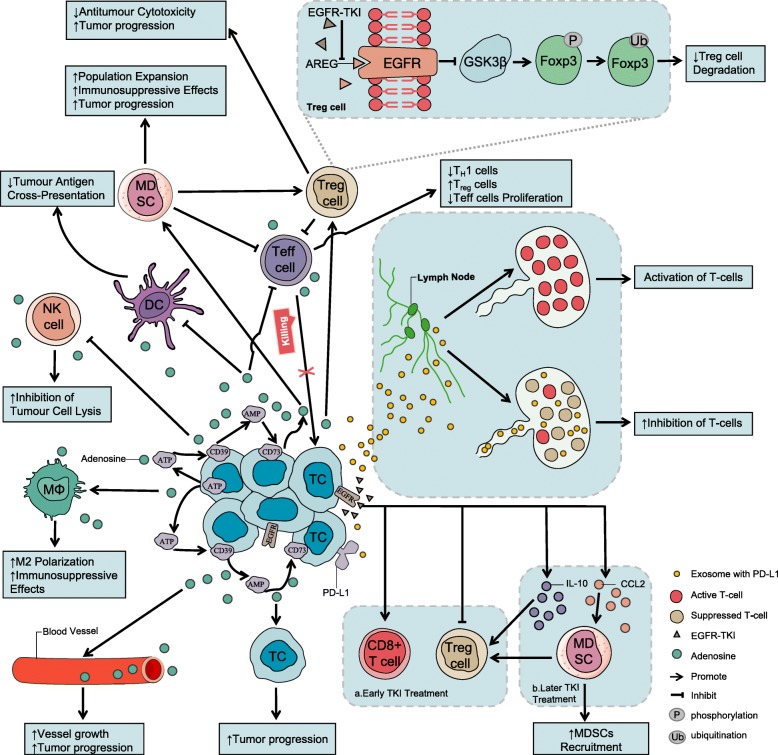
A major hallmark of immunosuppression in the TME through diverse pathways in EGFR-mutant NSCLC. EGFR-mutant tumor cells may upregulate CD73, convert ATP to ADO, which binds with subtypes of ADO receptors, and upregulate expression of Tregs by bypassing ADO, mediating tumor cell metastasis and proliferation. Abundant ADO exerts immunosuppressive activity on a variety of immune cells. It promotes activation of Tregs and accumulation of MDSCs, further attenuating antitumor function in NK and DC activity, skews Mφ polarization toward M2 macrophages and inhibits the Teff-mediated antitumor response, mediating tumor immunity escape. EGFR-TKIs alter immune profiles through the following pathways: enhancing expression of MHC (Fig. 2); promoting Foxp3 degradation to attenuate the inhibitory function of Tregs; reducing infiltration of Tregs in the TME and inhibiting tumor growth; and enhancing Teff-mediated antitumor activity, reducing T cell apoptosis, inhibiting M2-like polarization of macrophages and increasing levels of IL-10 and CCL2. CCL2 binds to its receptor CCR2 to act as a chemokine ligand, playing a critical role in the migration of MDSCs to the TME. In addition, CCL2 can upregulate and activate the STAT3 pathway of MDSCs. STAT3 further mediates the amplification and activation of MDSCs. MDSCs exert antitumor immunosuppressive actions, such as producing immunosuppressive molecules, inhibiting antitumor functions, inducing T cell apoptosis, and upregulating Tregs. However, EGFR-TKIs have a dynamic effect on the tumor immune microenvironment and modify the TME in several ways. AREG might regulate the efficiency of Treg-mediated immune modulation via the EGFR/GSK-3β/Foxp3 axis. GSK-3β-phosphorylated Foxp3 induces subsequent ubiquitination and degradation of Foxp3. Furthermore, loss of Foxp3 protein expression may be linked to impaired function of Tregs by affecting Foxp3 protein stability and its ability to bind to gene promoters. Exosomal PD-L1 suppresses T cell activity in draining lymph nodes (in mouse models). STAT3: signal transducer and transcriptional activator-3; CCL2: C-C motif chemokine ligand 2; Foxp3: forkhead box P3; NKs: natural killer cells; DCs: dendritic cells; Tregs: Treg cells; ADO: adenosine; Teffs: effector T cells; MHC: major histocompatibility complex; EGFR-TKIs: epidermal growth factor receptor tyrosine kinase inhibitors; MDSCs: myeloid-derived suppressor cells; IL-10: interleukin 10; GSK-3β: glycogen synthase kinase 3β; EGFR: epidermal growth factor receptor; TME: tumor microenvironment; CCR2: C-C motif chemokine receptor 2; ATP: adenosine triphosphate; PD-L1: programmed death-ligand 1; Tc: tumor cells; Mφ: macrophages; CD8+ T cells: cytotoxic T cells; TH1 cells: type 1 T helper cells

Immunosuppressive effects of EGFR mutations have also been described in recent years. Several studies have reported that EGFR mutations can modulate possible factors related to the status of the TME, such as tumor-infiltrating lymphocytes (TILs) [41, 43, 65], Tregs [45, 66], MDSCs [47, 67] tumor-associated macrophages (TAMs) [47], immunoregulatory cytokines [47, 48] and exosomes [68]. These preclinical and clinical findings suggest that the TME of NSCLC patients with EGFR mutations may be unique, differing from patients with wildtype EGFR, and that EGFR mutations may impact the antitumor immune response by affecting the TME (Fig. 1).

#### EGFR mutations and regulatory T cells (Tregs)

Tregs are regarded as a critical hurdle in antitumor immunity, and the transcriptional factor Foxp3 serves as a lineage specification factor for Tregs [69, 70]. TGF-β, IL-10 and IL-35 secreted by Tregs in tumors can produce an immunosuppressive environment that actively attenuates and subverts the antitumor immune responses of CD4+ T cells, CD8+ T cells and natural killer (NK) cells [71–74].

Huang et al. [66] showed that EGFR-containing exosomes induce the plasticity transformation of tolerant DCs and cause DCs to produce indoleamine 2, 3-dioxygenase (IDO), which plays an important role in converting CD3 + CD4 + CD25- T cells into Tregs; these results suggest that IDO expression can upregulate Treg function and induce immune tolerance and evasion [75, 76]. In addition, studies have shown that amphiregulin (AREG) is one of the EGFR ligands, and its level of plasma expression in patients with NSCLC is associated with a poor prognosis [77]; moreover, as a specific molecule in exosomes of tumor cells, AREG plays a role in promoting tumor progression [78]. Wang et al. [79] found that AREG meditated Treg suppressive function via the EGFR/GSK-3/Foxp3 axis in vitro and in vivo. Furthermore, inhibition of EGFR by the EGFR-TKI gefitinib restored the activity of GSK-3β and attenuated Treg function [80]. Mascia et al. [45] also showed that knockdown of the EGFR gene significantly inhibited tumor cell growth and downregulated Treg infiltration in the TME.

#### EGFR mutations and tumor-infiltrating lymphocytes (TILs)

TILs are a group of tumor-infiltrating and antigenic cell populations that can exist in tumor cancer nests and stroma [81]. CD8+ T cells act as antitumor immune cells during the development of the TME and destroy malignant T cells by releasing cytokines such as IFN-γ, perforin and granzyme B; the amount of CD8+ T cells determines the efficiency of tumor cell killing. Multiple studies have shown that highly infiltrating CD8+ TILs in NSCLC are associated with a good prognosis and good treatment efficacy [31, 82–84]. Teng et al. [85] evaluated the efficacy of immunotherapy by establishing a TME model based on the expression of TIL and PD-L1, suggesting that the immunoinflammatory TME (PD-L1+ and TIL+) is most likely to benefit from anti-PD-1/PD-L1 treatments and that lower levels of CD8+ TILs are associated with EGFR mutations [41, 43, 65, 86].

Dong et al. [43] found significantly reduced CD8+ TILs in an EGFR-mutant group compared with a wildtype EGFR group (*P* = 0.003). Notably, a significant difference in PD-L1 and CD8+ TIL combined expression between EGFR mutations showed a significantly lower ratio of PD-L1+/TIL+ but a higher ratio of PD-L1−/TIL- than the EGFR wildtype group (odds ratio (OR): 1.79, 95% CI: 1.10–2.93; *P* = 0.02). Investigators also found a significant difference in EGFR mutations between the PD-L1−/TIL- group and the PD-L1+/TIL+ group (*P =* 0.005), and patients with low PD-L1+/TIL+ carried EGFR mutations. Quantitative fluorescence images revealed TIL activation through identification of Ki67 (proliferation of T cells) and granzyme B (cytotoxic activity of TILs) in CD3+ cells [87]. Toki et al. [88] used fluorescence to explore the association between EGFR mutations and TIL status. An exhausted or dormant immune status (high CD3 with low Ki67 and low granzyme B) was detected in 28.6% (16/56) of those in the mutant EGFR group, and tumor cells and stromal cells with high PD-L1 expression were more likely to have highly infiltrating activated TILs (*P =* 0.0014 and *P =* 0.02, respectively). In addition, differences in immunological profiles according to EGFR mutation sites have been reported: the prevalence of the inflammatory TME, such as significantly higher expression of CD8+ T cells (*P =* 0.03) in EGFR L858R samples as well as a trend of higher levels of CD3+ and CD4+ T cells (*P =* 0.11 and *P =* 0.11, respectively) than in EGFR exon 19 deletion samples was observed; a higher level of infiltrating functional TILs was noted in the EGFR L858R group, but without differences in PD-L1 expression in tumor cells and stromal cells [88, 89].

#### EGFR mutations and exosomes

Exosomes are small membrane vesicles that are secreted by cells and contain many molecules, such as nucleic acids, lipids and proteins [89]. Exosomes act as a signal carrier to mediate cell-to-cell communication and affect the sensitivity of tumor cells to drugs, which is associated with the occurrence of tumor metastasis [72, 73, 90–96]. Tumor cell-derived exosomes can affect distant target cells through their intrinsic miRNAs, alter the local microenvironment, and form a pretransfer endometrium to exert remote regulatory functions [97]. Poggio et al. [68] reported that tumor cells secrete exosomes harboring PD-L1, leading to immune escape via direct binding to T cells and inhibition of their function. In addition, PD-L1-carrying exosomes are able to inhibit the activity of T cells in lymph nodes. It was also found that PD-L1 in exosomes is resistant to PD-L1 inhibitors and that knockout by clustered regularly interspaced short palindromic repeats (CRISPR) of genes related to exosomes can cause systemic antitumor immunity and immune memory and have significant effects after immunotherapy. In addition, the combined inhibition of exosome formation and anti-PD-1/PD-L1 treatment resulted in significantly longer survival time in mice compared to mice receiving monotherapy [68].

#### Cell surface molecules and selected soluble factors

Changes in expression of membranous immunomodulatory molecules in the TME and release of immunosuppressive soluble factors, such as TGF-β, IL-10 and adenosine (ADO) [47, 48], play a crucial role in tumor progression.

##### EGFR mutations and CD73

CD73 is an extracellular 5′-nucleotidase anchored to cell membrane lipid rafts by glycosylphosphatidylinositol (GPI), which is highly expressed in various tumors. CD73 is not only involved in purine and pyrimidine nucleotide synthesis and salvage pathways but is also an important negative regulator of immune signaling involved in the immune escape of tumors by catalyzing the formation of ADO, which is an immunosuppressive medium [98]. Studies have shown that high expression of CD73 is associated with both immunosuppression and poor prognosis in patients with NSCLC [98–100]. Therefore, understanding the crosstalk between CD73, ADO and TME is an area of active research, as described below (Fig. 1).

Park et al. [48] stratified data according to CD73 expression levels [CD73 high expression (CD73-H) and CD73 low expression (CD73-L)] and found that compared with the CD73-L group, the CD73-H group was less likely to have high-density infiltrating activated CD4+ T cells (20% vs 41%, *P <* 0.01) and CD8+ T cells (28% vs 47%, *P <* 0.01). The OS and median disease-free survival (DFS) were higher in the CD73-L group compared to the CD73-H group (62 vs 44 months, *P <* 0.01; 83 vs 34 months, *P <* 0.01), and subgroup findings suggested an association between EGFR mutations and higher CD73 expression (*P =* 0.03) [48]. Therefore, Park et al. hypothesized that overexpression of CD73 in mutant EGFR NSCLC may result in a poor response to immunosuppressive therapy and suggested that the combination of CD73 inhibitors with EGFR-TKIs or PD-1/PD-L1 inhibitors may be a potential strategy for treating drug-resistant patients [99]. In contrast, a retrospective study reported that CD73 overexpression compared to low CD73 expression in an EGFR-TKI-resistant group treated with immunotherapy resulted in a longer median PFS (16 months vs 1.2 months, *P =* 0.024) and ORR (66.7% vs 0%, *P =* 0.006), with no difference between in the high and low CD73 expression groups of wildtype EGFR patients (median PFS: 2.8 months vs 2.8 months, *P =* 0.394) [101]. However, the current consensus suggests that EGFR-mutant tumor cells may upregulate CD73, convert ATP to ADO, upregulate expression of Tregs through ADO bypass, and change the function of tumor cells and immune cells, resulting in an immunosuppressive TME. Due to inconsistent results, the precise mechanism by which CD73 expression is associated with an immunosuppressive TME remains unclear.

##### EGFR mutations and major histocompatibility complex (MHC)

**Fig. 2:**
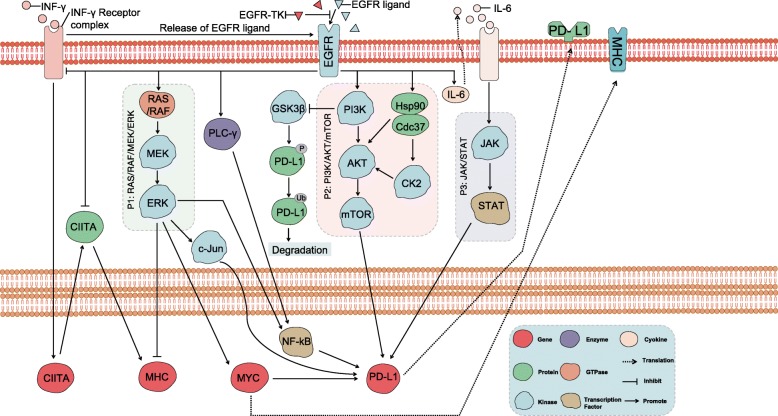
Intrinsic cancer cell pathways mediate the regulation of PD-L1 expression and MHC in EGFR-mutant NSCLC. Activation of EGFR may lead to downregulation of MHC expression through the MEK/ERK signaling pathway. In addition, activation of EGFR may influence expression of IFN-γR, generating intracellular signals that induce expression of the CIITA gene. CIITA is recruited to the MHC promoter, activating transcription. The net result is attenuation of CIITA and MHC molecule expression. In response to EGFR-TKIs, expression of CIITA and MHC genes is derepressed. In addition, EGFR-TKIs enhance MHC expression by inhibiting ERK activation. CIITA: class II major histocompatibility complex transactivator; TME: tumor microenvironment; MEK/ERK: extracellular signal–regulated kinase (ERK) kinase MEK; IFN-γR: interferon γ receptor; MHC: major histocompatibility complex; EGFR-TKIs: epidermal growth factor receptor tyrosine kinase inhibitors; EGFR: epidermal growth factor receptor; IFN-γ: interferon-γ

MHC plays an important role in tumor antigen presentation. MHC class I molecular tumor antigens constitute the first signal of cell activation, activate CD8+ T cells, and exert antitumor immune effects. MHC class II molecules bind to tumor antigen peptides and are presented to CD4+ T cells, which activate specific CD4+ T cells. The former can specifically kill tumor cells, and the latter participate in the body’s antitumor positive feedback regulation [102] by secreting cytokines to enhance the cell-killing effect. It has been previously reported that expression of MHCI and/or MHCII molecules can impact the antitumor immune response [103–105]. IFN-γ potentiates the induction of MHC class I (MHCI) and II (MHCII) molecules [102, 106]. Watanabe et al. found that EGFR-mutant cells have lower levels of human leukocyte antigen (HLA)-B expression than do EGFR-wildtype cells [107] in the presence of IFN-γ. Additionally, several recent studies report that MHC-I and MHC-II expression is downregulated via the IFN-γ signaling pathway and downstream MEK/ERK signaling pathways (Fig. 2) [102, 107–111].

### EGFR gene mutation sites and the effectiveness of immune checkpoint inhibitors

EGFR gene mutations mainly occur in exons 18–20; the exon19 deletion mutation (p. E746-A750del) and exon 21 point mutations (p.L858R) account for more than 85% of all mutation types. These two mutations are also the two [112] that result in greatest sensitivity to EGFR-TKIs. All other mutations can be referred to as uncommon mutations. The G719X mutation in exon 18 (3%) [113] and the L861X mutation in exon 21 (2%) [114] are the most common uncommon mutation types; G719X, L861X, and additional mutations in exon 20 and exon 19 are also considered favorable for effective EGFR-TKI treatment [115–117]. Recently, multiple studies have shown that NSCLC patients with uncommon EGFR mutations are more likely to benefit from immunotherapy than are those with common EGFR mutations [118, 119].

Yamada et al. [118] reported that NSCLC patients with uncommon EGFR mutations showed a good response to ICIs compared with patients with common EGFR mutations, with prolonged median PFS (256 days vs 50 days, *P =* 0.003) and median time to progression (TTP) (256 days vs 48 days, *P =* 0.008). A 5-year follow-up of the CA209–003 study on nivolumab, a phase I single-arm study, was reported by Brahmer and colleagues [120], who observed that 2 patients with uncommon EGFR mutations (2/8, 25%) had a survival of more than 5 years; the mutations were the EGFR exon 20 insertion and exon 18 missense mutation G719A. Thus far, long-term efficacy and safety data for ICIs from randomized trials have been lacking for NSCLC. Additionally, in a report by Yoshida and colleagues [119], a longer PFS rate was observed in patients with uncommon EGFR mutations treated with nivolumab than in those with common EGFR mutations (*P* < 0.05) [47].

### EGFR mutations and tumor mutation burden (TMB)

The TMB is the total number of substitution, insertion, and deletion mutations per megabase of the coding region of a tumor gene; it is a good biomarker for predicting the efficacy of immunotherapy and can quantitatively estimate the total number of mutations in the coding region of the tumor genome. A higher TMB is related to more new antigens produced by tumors, easier recognition by immune cells and a long-lasting clinical response [38].

Notably, compared to an EGFR-resistant/unknown group, a significantly lower TMB was found for EGFR-sensitive mutations (defined based on response to first-generation EGFR-TKIs. Haratani et al. [86] evaluated the efficacy of nivolumab with regard to TMB in NSCLC patients with mutant EGFR; a median TMB of 101 was reported for each tumor patient, and patients who dramatically responded to nivolumab had a significantly higher TMB than did non-responders. In addition, Dong et al. [43] found a significantly reduced median TMB in an EGFR-mutant group (exons19Del, L858R, L861Q, G719X, and S768I) compared with an EGFR-wildtype group (56 vs 181). They also found that the median ratio of the EGFR mutant to wildtype TMB was 59:209 (Broad data set) and 162:197 (GLCI data set). Mutations such as those in EGFR, BRAF and TP53 have been shown to typically occur as early clonal, initiating drivers. Current studies show that most patients with specific gene mutations do not respond well to immunotherapy, and reduced TMB may be a mechanism for a poor response to ICIs in patients with EGFR mutation [37, 43, 49]. Attempts to establish a correlation between EGFR mutations and TMB are ongoing, and the role of microsatellite instability-high (MSI-H) and mismatch-repair deficiency (dMMR) in immunotherapy also needs to be explored.

### EGFR mutations and PD-L1 expression

The regulation of EGFR mutations and PD-L1 expression remains controversial, but experimental data [121–124] indicate that EGFR mutations directly or indirectly drive PD-L1 upregulation in NSCLC cells (co-cultured with immune cells). In addition, there is some evidence to support that activation of downstream EGFR signaling [124–134], such as Ras/RAF/MEK/ERK, PI3K/AKT/mTOR, JAK/STAT, NF-kB and GSK-3β, leads to PD-L1 expression (Fig. 2). However, this “intrinsic” mechanism of PD-L1 upregulation is contrasted by several recent clinical studies concluding that PD-L1 is highly expressed in EGFR-wildtype NSCLC [135], and there is a negative correlation [43, 136–139] or no significant correlation [135] between EGFR mutations and PD-L1 expression. One meta-analysis [139] revealed lower PD-L1 expression rates in EGFR-mutant than in EGFR-wildtype tumors (36.7% vs 44.1%, *P <* 0.05). Another pooled analysis [43] had the same conclusion: expression of PD-L1 in EGFR-wildtype tumors was significantly higher than that in EGFR-mutant tumors (*P =* 0.02). For further confirmation, the researchers examined mRNA profiles, PD-L1 immunohistochemistry (IHC) and reverse-phase protein arrays (RPPAs) of tumor samples and found that expression of PD-L1 in EGFR-mutant tumors was significantly lower than that in the EGFR-wildtype tumors (*P <* 0.05). Nonetheless, others have reported the opposite results [51, 122].

Such inconsistent results of different experimental studies might be associated with various factors, such as different PD-L1 detection techniques (different antibodies, detection platforms, and different set positive thresholds), tumor heterogeneity, and patient tumor tissue sources (such as cytological specimens, archived specimens, fresh specimens, primary and metastasis sites). Additionally, increased expression of PD-L1 on immune cells can induce immune escape. TILs can also be used to detect PD-L1 expression [20, 140–145]. Noguchi et al. [146] reported that expression of PD-L1 on TAMs might have an important role in tumor immune escape. Induction of PD-L1 on tumor cells is regulated by two major pathways: one driven by IFN-γ and another controlled by constitutive oncogenic signaling. However, as expression of PD-L1 on immune cells is pronounced, only partially dependent on IFN-γ, and is relatively stable during monitoring, Noguchi et al. concluded that expression of PD-L1 on immune cells is a good biomarker [146].

### T790 M mutation status and the effectiveness of immune checkpoint inhibitors

An EGFR-sensitive mutation group (defined based on response to first-generation EGFR-TKIs) exhibited a significantly longer PFS compared to an EGFR-resistant/unknown group [147–150]. However, acquired resistance to EGFR-TKIs develops after 9–14 months, and approximately 50–60% of such resistance is mediated by T790 M [151]. Haratani et al. [86] observed a benefit of nivolumab in T790 M(−) patients compared with T790 M(+) patients (median PFS: 2.1 months vs 1.3 months). Although the number of CD8+ TILs in the T790 M(+) and T790 M(−) patients with EGFR mutations was similar, the proportion of tumors with PD-L1 level ≥ 10% or ≥ 50% (20% vs 4%) and high-density CD8+ TILs (≥median) (12% vs 4%) was higher among T790 M(−) patients. Additionally, T790 M(−) patients had significantly lower FOXP3+ TILs than did T790 M(+) patients (*P =* 0.013). Furthermore, in a retrospective study by Yamada et al. [118] including 27 patients with EGFR-TKI resistance who were treated with ICIs, subgroup findings supported that T790 M(−) patients were more likely to derive greater benefit from PD-1 inhibitor treatment (median PFS: 86 days vs 48 days, *P =* 0.03; median TTP: 97 days vs 48 days, *P =* 0.03) than were T790 M(+) patients. In the study of 67 EGFR-mutant NSCLC patients, the prevalence of PD-L1 expression was significantly lower in T790 M(+) tumors than in T790 M(−) tumors (*P =* 0.0149), and better survival was associated with PD-L1(−) / T790 M(+) tumors [152].

## Immune modulatory effects of EGFR-TKIs

### EGFR-TKIs affect the TME in NSCLC

To date, preclinical and clinical studies have shown that EGFR-TKIs can induce antitumor immunity through the following [45, 79, 102, 107, 108, 153–156]: potentiating induction of class I (MHCI) and II (MHCII) molecules; promoting Foxp3 degradation to attenuate the inhibitory function of Tregs; reducing the infiltration of Tregs in the TME and inhibiting tumor growth; and enhancing the cytotoxicity of cytotoxic T lymphocytes (CTLs) that mediate antitumor immune response, reduce T cell apoptosis, and increase IFN-γ secretion to enhance the immune system response. Thus, EGFR-TKIs show promising efficacy for anti-PD-1/PD-L1 treatment. However, these possible mechanisms regarding the immunostimulatory effect of EGFR-TKIs do not fully explain the controversial results of the combination of ICIs and EGFR-TKIs in patients with EGFR mutations [27, 28, 30].

Using a murine model, Jia et al. [47] recently observed a dynamic effect of EGFR-TKIs on the tumor immune microenvironment from beneficial (early treatment) to immunosuppressive (later treatment) (Fig. 1; Table 2). The short-term inhibition of tumor cell growth early in EGFR-TKI treatment is obvious, including an increase in the numbers of CD8+ T cells, DCs and M1-like TAMs, a decrease in Treg infiltration and inhibition of M1-like TAMs to M2-like TAMs. Jia and colleagues also reported that certain immunosuppressive factors accumulate gradually throughout treatment. However, later in EGFR-TKI treatment, they found there was either no significant change or even a decrease in antitumor effector cells and increasing secretion of IL-10 and CCL2 in serum. CCL2, a key effector cytokine with expression that is upregulated by EGFR-TKIs, plays an important role in the migration of MDSCs to the TME [157–160]. CCL2 induces T cells to differentiate to Th2 cells (anti-inflammatory function), which upregulate and activate the signal transducer and transcriptional activator-3 (STAT3) pathway of MDSCs. Thus, STAT3 further mediates the amplification and activation of MDSCs [161], exerting antitumor immunosuppressive effects [162], such as producing the immunosuppressive molecules IL-10 and TGF-β, inhibiting antitumor functions [163–168], inducing T cell apoptosis [169], upregulating Tregs [170] and promoting M2 phenotype polarization in TAMs [171]. In addition to suppressing immune responses, MDSCs are associated with tumorigenesis by promoting metastasis and inducing angiogenesis, including the secretion of vascular endothelial growth factor (VEGF), direct differentiation into tumor vascular endothelial cells [172], and release of matrix metalloproteinase (MMP) [173, 174]. Investigators have also reported that IL-10 [175] not only mediates immature myeloid cells (IMCs) via the STAT3 pathway to activate MDSCs but also suppresses HLA class expression on the tumor cell surface. Thus, according to the data by Jia and colleagues, there may be a small window of immune microenvironmental changes in which EGFR blockade is most beneficial in the setting of combinations with immune-mediated anticancer approaches. One key question is whether a similar phenomenon occurs in EGFR-TKI-treated NSCLC, which is critical to improve the efficacy of immunotherapy combined with targeted therapy.

**Table 2:** The Dynamic Changes Occurring in the Immune Microenvironment after EGFR Inhibition in an EGFR-Mutant Transgenic Mouse Model

Effect of EGFR-TKIs on tumor immune Microenvironment	Early Treatment	Later Treatment	Throughout Treatment
Antitumor Activity	Tumor burden↓	Tumor burden↓	Tumor burden↓
	Tumor size↓	Tumor size↓	Tumor size↓
Expression of Immune Checkpoint Molecules	PD-L1↓^a^	PD-L1↓^a^	PD-L1↓^a^
	PD-1↓^b^	PD-1↓^b^	PD-1↓^b^
	CTLA-4↓^b^	CTLA-4↓ ^b^	CTLA-4↓^b^
	TIM-3↓^b^	TIM-3↓^b^	TIM-3↓^b^
Lymphocyte Infiltration	CD3+ lymphocytes ↑	CD3+ lymphocytes +/−	
	CD8+ T cells↑	CD8+ T cells	
	Foxp3+ Tregs↓	Foxp3+ Tregs	
Macrophage Infiltration	CD11b + Myeloid Cells↑	CD11b + Myeloid Cells↑	CD11b + Myeloid Cells↑
	PMN-MDSCs +/−	PMN-MDSCs +/−	PMN-MDSCs +/−
	M-MDSCs↑	M-MDSCs↑	M-MDSCs↑
	DCs↑	DCs+/−	
	M1-TAM↑	M1-TAM↓	
	M2-TAM↓	M2-TAM↓	M2-TAM↓
Cytokine Secretion	IL-10 +/−	IL-10↑	
	CCL-2 +/−	CCL-2↑	

*PD-L1* Programmed death-ligand 1, *PD-1* Programmed cell death protein 1, *CTLA-4* The cytotoxic T-lymphocyte–associated antigen 4, *TIM-3* T-cell immunoglobulin and mucin-domain containing-3, *PMN-MDSCs* Polymorphonuclear MDSCs, *M-MDSCs* Mononuclear MDSCs, *IL-10* Interleukin 10, *CCL-2* The chemokine (C- C motif) ligand 2

^a^: PD-L1 expression after EGFR-TKI monotherapy in both CD45+ immune cells and CD4 − tumor cells;

^b^: PD-1, CTLA-4, and TIM-3 expression on CD3+ lymphocytes;

+/−: Remained unchanged

↑: increased

↓: decreased

### EGFR-TKIs cause dynamic changes in expression of PD-L1

An intriguing phenomenon occurs in EGFR-TKI-treated NSCLC. Studies have shown that PD-L1 expression is dynamic during the course of EGFR-TKI treatment, EGFR-TKIs might repress PD-L1 expression, and PD-L1 expression is increased following EGFR-TKI treatment [41, 176]. The significant increase in PD-L1 expression in some patients may explain some of the better outcomes of second-line immunotherapy in patients with EGFR-TKI resistance. Notably, PD-L1 is upregulated in a subset of patients with NSCLC harboring EGFR mutations and is associated with primary resistance to EGFR-TKIs, with reported incidences ranging from 21 to 38.9% [41, 176–178]. As reported by Hsu and colleagues, a PD-L1 Tumor Proportion Score (TPS) ≧50% for clinical NSCLC specimens was associated with a significant risk of acquiring primary resistance to EGFR-TKIs when compared to patients with PD-L1 TPS < 50%, with an odds ratio (OR) of 16.47 (95% Cl: 2.10–129.16, *P =* 0.008) in a study of 66 surgically resected samples [177]. Gainor and colleagues [41] detected the level of PD-L1 in paired tumor tissues before EGFR-TKI treatment and tissues after development of resistance to EGFR-TKIs, and the results showed marked increases in PD-L1 expression in 12 patients (21%). Another study [176] reported that PD-L1 expression was increased in 7 patients (38.9%) with development of resistance to gefitinib, with high mesenchymal–epithelial transition (MET) activity (*P =* 0.028). In vitro data also showed that PD-L1 expression is upregulated in cells resistant to gefitinib. Nevertheless, the underlying mechanism by which PD-L1 expression is associated with primary resistance to EGFR-TKIs remains unclear.

## Future prospects

**Fig. 3:**
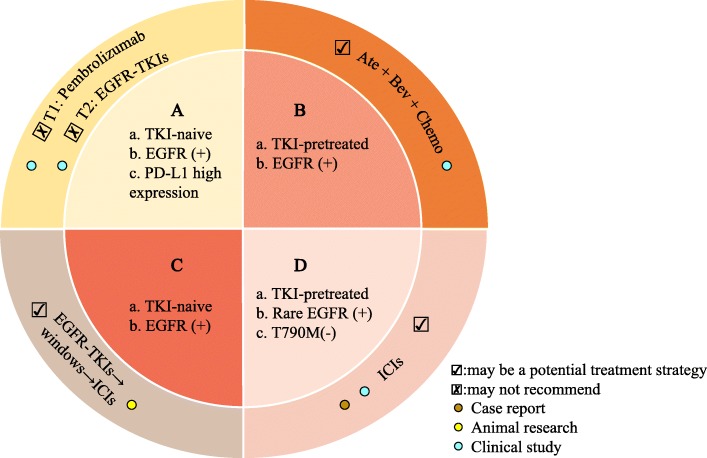
Representative tumor characteristics and treatment regimens for NSCLC patients with EGFR mutations. TKI: tyrosine kinase inhibitors; EGFR: epidermal growth factor receptor; PD-L1: programmed death-ligand 1; ICIs: immune checkpoint inhibitors; T1: treatment 1; T2: treatment 2; Ate: atezolizumab; Bev: bevacizumab; Chemo: chemotherapy; (+): positive; (−): negative

The application of EGFR-TKIs in combination with ICIs in routine clinical practice for patients carrying EGFR mutations has raised several concerns that have yet to be resolved by the multiple clinical trials conducted to date. Notably, traditional lung cancer treatments are far simpler than the present situation demands. After failure of first-line TKIs, patients with EGFR mutations have limited treatment options [12, 179]. Thus, there is an urgent need to further investigate novel treatment strategies. This review summarizes some potential benefits in this regard. First, administering pembrolizumab prior to EGFR-TKI treatment is not recommended as the first-line treatment for EGFR-TKI-naïve, high PD-L1-expressing tumors, which is supported by a phase II trial (NCT0287994) [180]. The data showed that no EGFR-mutant NSCLC patients received a benefit from pembrolizumab administration prior to EGFR-TKI treatment [180]. Second, ICIs may be a promising approach for some cases with high PD-L1 expression or some uncommon EGFR-mutated NSCLC cases due to intratumor heterogeneity among PD-L1-expressing and EGFR-mutant clones [30, 118–120]. Third, tumor microenvironmental changes, a rather small window, may be most beneficial for combination EGFR blockade with immune-mediated anticancer approaches, which were found to only be temporary and disappeared as treatment continued [47]. Fourth, EGFR-TKIs may not be optimal for EGFR-TKI-naïve, high PD-L1 expressing, EGFR-mutated NSCLC as the first-line treatment due to a possible association between high PD-L1 expression and primary resistance to EGFR-TKIs [177, 178]. Fifth, the combination of VEGF/VEGFR inhibitors with ICIs may represent a new option for patients with EGFR mutations for whom TKIs have failed. Several possible mechanisms regarding immune-modulatory effects [181] through VEGF inhibition have been proposed, including T cell priming promotion and activation via DC maturation [181, 182], increased T cell tumor infiltration via normalization of the tumor vasculature through VEGF inhibition [183–185], and establishment of an immune-permissive TME via decreases in MDSC and Treg populations [181, 185]. However, the precise mechanisms of VEGF blockade and immune modulatory effects remain unclear. One randomized phase III trial (IMpower150) was conducted to test the efficacy of adding atezolizumab to standard-of-care bevacizumab and chemotherapy in NSCLC patients carrying EGFR mutations, and intriguingly, this approach has shown promising efficacy (median DOR: 11.1 months vs 5.6 months) compared with standard-of-care bevacizumab and chemotherapy [186]. Sixth, most EGFR-mutant advanced NSCLC patients harbor multiple co-occurring oncogenic mutations; thus, genomic molecular diagnosis should be applied to further select the most appropriate treatment strategy [187]. Given this complexity, it is essential to identify the optimal sequence of treatment and strategies for NSCLC patients with EGFR mutations (Fig. 3).

## Conclusion

Globally, lung cancer has the highest rates of diagnosis and mortality among cancers. NSCLC accounts for more than 85% of all lung cancer cases, seriously threatening human health. Currently, immunotherapy is a very promising therapeutic strategy for NSCLC. Preclinical studies indicate that EGFR mutations mediate tumor immune escape through the PD-1/PD-L1 pathway and that EGFR-TKIs downregulate PD-L1 expression [121, 125–130]. Overall, NSCLC Patients with EGFR mutations do not respond well to immunotherapy. However, some studies have shown that immunotherapy is still effective in patients with EGFR mutations. This review summarizes the current status of NSCLC patients with EGFR mutations who are treated with immunotherapy alone or in combination with EGFR-TKIs. During treatment with EGFR-TKIs, EGFR mutations may result in dynamic changes in the immunological profile, such as a dynamic immune TME, a low TMB, and altered expression of PD-L1. These contradictions and controversies suggest that immunotherapy or EGFR-TKI combination therapy in NSCLC patients with EGFR mutations requires steps of clinical validation and utility. Therefore, in this case, mechanisms to induce long-lasting antitumor activity in the TME and to maximize the effect of immunotherapy in patients can still be improved. There is also a clear unmet need for establishing prognostic molecular and clinical markers, dosages, schedules, the optimal sequence of treatment and strategies when combining immunotherapy with other therapies. We believe that the long-term survival of NSCLC patients with EGFR mutations may be very promising.

## Data Availability

Not applicable.

## Abbreviations

(−): Negative
(+): Positive
ADO: Adenosine
Ate: Atezolizumab
ATP: Adenosine triphosphate
Bev: Bevacizumab
CCL2: C-C motif chemokine ligand 2
CCR2: C-C motif chemokine receptor 2
CD8+ T cells: Cytotoxic T cells
Chemo: Chemotherapy
CIITA: Class II major histocompatibility complex transactivator
CTLA-4: Cytotoxic T-lymphocyte–associated antigen 4
DCs: Dendritic cells
EGFR: Epidermal growth factor receptor
EGFR-TKIs: Epidermal growth factor receptor tyrosine kinase inhibitors
Foxp3: Forkhead box P3
GSK-3β: Glycogen synthase kinase 3β
IFN-γ: Interferon-γ
IFN-γR: Interferon γ receptor
IL-10: Interleukin 10
MDSCs: Myeloid-derived suppressor cells
MEK/ERK: Extracellular signal-regulated kinase (ERK) kinase MEK
MHC: Major histocompatibility complex
M-MDSCs: Mononuclear MDSCs
Mφ: Macrophages
NKs: Natural killer cells
NSCLC: Non-small cell lung cancer
ORR: Objective response rate
OS: Overall survival
PD-L1: Programmed death-ligand 1
PFS: Progression-free survival
PMN-MDSCs: Polymorphonuclear MDSCs
STAT3: Signal transducer and transcriptional activator-3
Tc: Tumor cells
Teffs: Effector T cells
TH1 cells: Type 1 T helper cells
TIM-3: T cell immunoglobulin and mucin-domain containing-3
TME: Tumor microenvironment
Tregs: Treg cells

## References

[1] Bray F, Ferlay J, Soerjomataram I, Siegel RL, Torre LA, Jemal A (2018). Global cancer statistics 2018:GLOBOCAN estimates of incidence and mortality worldwide for 36 cancers in 185 countries. CA Cancer J Clin.

[2] Torre LA, Bray F, Siegel RL, Ferlay J, Lortet-Tieulent J, Jemal A (2015). Global cancer statistics, 2012. CA Cancer J Clin.

[3] Torre LA, Siegel RL, Jemal A (2016). Lung cancer statistics. In Lung cancer and personalized medicine.

[4] Taylor MD, LaPar DJ, Isbell JM, Kozower BD, Lau CL, Jones DR (2014). Marginal pulmonary function should not preclude lobectomy in selected patients with non–small cell lung cancer. J Thorac Cardiovasc Surg.

[5] Chen Z, Fillmore CM, Hammerman PS, Kim CF, Wong KK (2014). Non-small-cell lung cancers: a heterogeneous set of diseases. Nat Rev Cancer.

[6] Remon J, Hendriks LE, Cabrera C, Reguart N, Besse B (2018). Immunotherapy for oncogenic-driven advanced non-small cell lung cancers:is the time ripe for a change?. Cancer Treat Rev.

[7] Hanna N, Johnson D, Temin S, Baker S, Brahmer J, Ellis PM, Giaccone G, Hesketh PJ, Jaiyesimi I, Leighl NB, Riely GJ (2017). Systemic therapy for stage IV non-small-cell lung cancer:American Society of Clinical Oncology clinical practice guideline update. J Clin Oncol.

[8] Ettinger DS, Wood DE, Akerley W, Bazhenova LA, Borghaei H, Camidge DR, Cheney RT, Chirieac LR, D’Amico TA, Dilling TJ, Dobelbower MC (2016). NCCN guidelines insights:non–small cell lung cancer, version 4.2016. J Natl Compr Canc Netw.

[9] Wu YL, Saijo N, Thongprasert S, Yang JH, Han B, Margono B, Chewaskulyong B, Sunpaweravong P, Ohe Y, Ichinose Y, Yang JJ (2017). Efficacy according to blind independent central review:post-hoc analyses from the phase III, randomized, multicenter, IPASS study of first-line gefitinib versus carboplatin/paclitaxel in Asian patients with EGFR mutation-positive advanced NSCLC. Lung Cancer.

[10] Ohashi K, Maruvka YE, Michor F, Pao W (2013). Epidermal growth factor receptor tyrosine kinase inhibitor–resistant disease. J Clin Oncol.

[11] Helena AY, Arcila ME, Rekhtman N, Sima CS, Zakowski MF, Pao W, Kris MG, Miller VA, Ladanyi M, Riely GJ (2013). Analysis of tumor specimens at the time of acquired resistance to EGFR-TKI therapy in 155 patients with EGFR-mutant lung cancers. Clin Cancer Res.

[12] Mok TS, Wu YL, Ahn MJ, Garassino MC, Kim HR, Ramalingam SS, Shepherd FA, He Y, Akamatsu H, Theelen WS, Lee CK (2017). Osimertinib or platinum–pemetrexed in EGFR T790M–positive lung cancer. N Engl J Med.

[13] Chen DS, Mellman I (2013). Oncology meets immunology:the cancer-immunity cycle. Immunity.

[14] Lee CK, Man J, Lord S, Cooper W, Links M, Gebski V, Herbst RS, Gralla RJ, Mok T, Yang JC (2018). Clinical and molecular characteristics associated with survival among patients treated with checkpoint inhibitors for advanced non–small cell lung carcinoma:a systematic review and meta-analysis. JAMA Oncol.

[15] Brahmer J, Reckamp KL, Baas P, Crinò L, Eberhardt WE, Poddubskaya E, Antonia S, Pluzanski A, Vokes EE, Holgado E, Waterhouse D (2015). Nivolumab versus docetaxel in advanced squamous-cell non–small-cell lung cancer. N Engl J Med.

[16] Borghaei H, Paz-Ares L, Horn L, Spigel DR, Steins M, Ready NE, Chow LQ, Vokes EE, Felip E, Holgado E, Barlesi F (2015). Nivolumab versus docetaxel in advanced nonsquamous non–small-cell lung cancer. N Engl J Med.

[17] Herbst RS, Baas P, Kim DW, Felip E, Pérez-Gracia JL, Han JY, Molina J, Kim JH, Arvis CD, Ahn MJ, Majem M (2016). Pembrolizumab versus docetaxel for previously treated, PD-L1-positive, advanced non-small-cell lung cancer (KEYNOTE-010):a randomised controlled trial. Lancet.

[18] Rittmeyer A, Barlesi F, Waterkamp D, Park K, Ciardiello F, Von Pawel J, Gadgeel SM, Hida T, Kowalski DM, Dols MC, Cortinovis DL (2017). Atezolizumab versus docetaxel in patients with previously treated non-small-cell lung cancer (OAK):a phase 3, open-label, multicentre randomised controlled trial. Lancet.

[19] Fehrenbacher L, Spira A, Ballinger M, Kowanetz M, Vansteenkiste J, Mazieres J, Park K, Smith D, Artal-Cortes A, Lewanski C, Braiteh F (2016). Atezolizumab versus docetaxel for patients with previously treated non-small-cell lung cancer (POPLAR):a multicentre, open-label, phase 2 randomised controlled trial. Lancet.

[20] Reck M, Rodríguez-Abreu D, Robinson AG, Hui R, Csőszi T, Fülöp A, Gottfried M, Peled N, Tafreshi A, Cuffe S, O’Brien M (2016). Pembrolizumab versus chemotherapy for PD-L1–positive non–small-cell lung cancer. N Engl J Med.

[21] Carbone DP, Reck M, Paz-Ares L, Creelan B, Horn L, Steins M, Felip E, van den Heuvel MM, Ciuleanu TE, Badin F, Ready N (2017). First-line nivolumab in stage IV or recurrent non–small-cell lung cancer. N Engl J Med.

[22] Bianco A, Malapelle U, Rocco D, Perrotta F, Mazzarella G (2018). Targeting immune checkpoints in non small cell lung cancer. Curr Opin Pharmacol.

[23] William WN, Lin HY, Lee JJ, Lippman SM, Roth JA, Kim ES (2009). Revisiting stage IIIB and IV non-small cell lung cancer:analysis of the surveillance, epidemiology, and end results data. Chest.

[24] Siegel RL, Miller KD, Jemal A (2016). Cancer statistics, 2016. CA Cancer J Clin.

[25] Soo RA, Lim SM, Syn NL, Teng R, Soong R, Mok TS, Cho BC (2018). Immune checkpoint inhibitors in epidermal growth factor receptor mutant non-small cell lung cancer:current controversies and future directions. Lung Cancer.

[26] Li X, Lian Z, Wang S, Xing L, Yu J (2018). Interactions between EGFR and PD-1/PD-L1 pathway:implications for treatment of NSCLC. Cancer Lett.

[27] Yang JC, Shepherd FA, Kim DW, Lee GW, Lee JS, Chang GC, Lee SS, Wei YF, Lee YG, Laus G, Collins B (2019). Osimertinib plus durvalumab versus osimertinib monotherapy in EGFR T790M-positive NSCLC following previous EGFR-TKI therapy:CAURAL brief report. J Thorac Oncol.

[28] Yang JC, Gadgeel SM, Sequist LV, Wu CL, Papadimitrakopoulou VA, Su WC, Fiore J, Saraf S, Raftopoulos H, Patnaik A (2019). Pembrolizumab in combination with Erlotinib or Gefitinib as first-line therapy for advanced NSCLC with sensitizing EGFR mutation. J Thorac Oncol.

[29] Yi L, Fan J, Qian R, Luo P, Zhang J (2019). Efficacy and safety of osimertinib in treating EGFR-mutated advanced NSCLC:a meta-analysis. Int J Cancer.

[30] Antonia SJ, Brahmer JR, Gettinger S, Chow LQ, Juergens R, Shepherd FA, Laurie SA, Gerber DE, Goldman J, Shen Y, Harbison C (2014). Nivolumab (anti-PD-1; BMS-936558, ONO-4538) in combination with platinum-based doublet chemotherapy (PT-DC) in advanced non-small cell lung cancer (NSCLC):metastatic non-small cell lung cancer. Int J Radiat Oncol Biol Phys.

[31] Kamphorst AO, Wieland A, Nasti T, Yang S, Zhang R, Barber DL, Konieczny BT, Daugherty CZ, Koenig L, Yu K, Sica GL (2017). Rescue of exhausted CD8 T cells by PD-1–targeted therapies is CD28-dependent. Science.

[32] Overacre-Delgoffe AE, Chikina M, Dadey RE, Yano H, Brunazzi EA, Shayan G, Horne W, Moskovitz JM, Kolls JK, Sander C, Shuai Y (2017). Interferon-γ drives Treg fragility to promote anti-tumor immunity. Cell.

[33] Tavazoie MF, Pollack I, Tanqueco R, Ostendorf BN, Reis BS, Gonsalves FC, Kurth I, Andreu-Agullo C, Derbyshire ML, Posada J, Takeda S (2018). LXR/ApoE activation restricts innate immune suppression in cancer. Cell.

[34] Gebhardt C, Sevko A, Jiang H, Lichtenberger R, Reith M, Tarnanidis K, Holland-Letz T, Umansky L, Beckhove P, Sucker A, Schadendorf D (2015). Myeloid cells and related chronic inflammatory factors as novel predictive markers in melanoma treatment with ipilimumab. Clin Cancer Res.

[35] De Henau O, Rausch M, Winkler D, Campesato LF, Liu C, Cymerman DH, Budhu S, Ghosh A, Pink M, Tchaicha J, Douglas M (2016). Overcoming resistance to checkpoint blockade therapy by targeting PI3Kγ in myeloid cells. Nature.

[36] Kaneda MM, Messer KS, Ralainirina N, Li H, Leem CJ, Gorjestani S, Woo G, Nguyen AV, Figueiredo CC, Foubert P, Schmid MC (2016). PI3Kγ is a molecular switch that controls immune suppression. Nature.

[37] Rizvi NA, Hellmann MD, Snyder A, Kvistborg P, Makarov V, Havel JJ, Lee W, Yuan J, Wong P, Ho TS, Miller ML (2015). Mutational landscape determines sensitivity to PD-1 blockade in non–small cell lung cancer. Science.

[38] Yarchoan M, Hopkins A, Jaffee EM (2017). Tumor mutational burden and response rate to PD-1 inhibition. N Engl J Med.

[39] McGranahan N, Furness AJ, Rosenthal R, Ramskov S, Lyngaa R, Saini SK, Jamal-Hanjani M, Wilson GA, Birkbak NJ, Hiley CT, Watkins TB (2016). Clonal neoantigens elicit T cell immunoreactivity and sensitivity to immune checkpoint blockade. Science.

[40] Peng W, Chen JQ, Liu C, Malu S, Creasy C, Tetzlaff MT, Xu C, McKenzie JA, Zhang C, Liang X, Williams LJ (2016). Loss of PTEN promotes resistance to T cell–mediated immunotherapy. Cancer Discov.

[41] Gainor JF, Shaw AT, Sequist LV, Fu X, Azzoli CG, Piotrowska Z, Huynh TG, Zhao L, Fulton L, Schultz KR, Howe E (2016). EGFR mutations and ALK rearrangements are associated with low response rates to PD-1 pathway blockade in non–small cell lung cancer:a retrospective analysis. Clin Cancer Res.

[42] Havel JJ, Chowell D, Chan TA (2019). The evolving landscape of biomarkers for checkpoint inhibitor immunotherapy. Nat Rev Cancer.

[43] Dong ZY, Zhang JT, Liu SY, Su J, Zhang C, Xie Z, Zhou Q, Tu HY, Xu CR, Yan LX, Li YF (2017). EGFR mutation correlates with uninflamed phenotype and weak immunogenicity, causing impaired response to PD-1 blockade in non-small cell lung cancer. Oncoimmunology.

[44] Li HY, McSharry M, Bullock B, Nguyen TT, Kwak J, Poczobutt JM, Sippel TR, Heasley LE, Weiser-Evans MC, Clambey ET, Nemenoff RA (2017). The tumor microenvironment regulates sensitivity of murine lung tumors to PD-1/PD-L1 antibody blockade. Cancer Immunol Res.

[45] Mascia F, Schloemann DT, Cataisson C, McKinnon KM, Krymskaya L, Wolcott KM, Yuspa SH (2016). Cell autonomous or systemic EGFR blockade alters the immune-environment in squamous cell carcinomas. Int J Cancer.

[46] Saxon JA, Sherrill TP, Polosukhin VV, Sai J, Zaynagetdinov R, McLoed AG, Gulleman PM, Barham W, Cheng DS, Hunt RP, Gleaves LA (2016). Epithelial NF-κB signaling promotes EGFR-driven lung carcinogenesis via macrophage recruitment. Oncoimmunology.

[47] Jia Y, Li X, Jiang T, Zhao S, Zhao C, Zhang L, Liu X, Shi J, Qiao M, Luo J, Liu S (2019). EGFR-targeted therapy alters the tumor microenvironment in EGFR-driven lung tumors: implications for combination therapies. Int J Cancer.

[48] Park Lee Chun, Rhee Kyunghoon, Kim Won Bin, Cho Anderson, Song Junho, Anker Jonathan Forrest, Oh Michael, Bais Preeti, Namburi Sandeep, Chuang Jeffrey, Chae Young Kwang (2018). Immunologic and clinical implications of CD73 expression in non-small cell lung cancer (NSCLC). Journal of Clinical Oncology.

[49] Spigel David R., Schrock Alexa Betzig, Fabrizio David, Frampton Garrett Michael, Sun James, He Jie, Gowen Kyle, Johnson Melissa Lynne, Bauer Todd Michael, Kalemkerian Gregory Peter, Raez Luis E., Ou Sai-Hong Ignatius, Ross Jeffrey S., Stephens Philip J., Miller Vincent A., Ali Siraj Mahamed (2016). Total mutation burden (TMB) in lung cancer (LC) and relationship with response to PD-1/PD-L1 targeted therapies. Journal of Clinical Oncology.

[50] Akbay EA, Koyama S, Carretero J, Altabef A, Tchaicha JH, Christensen CL, Mikse OR, Cherniack AD, Beauchamp EM, Pugh TJ, Wilkerson MD (2013). Activation of the PD-1 pathway contributes to immune escape in EGFR-driven lung tumors. Cancer Discov.

[51] Azuma K, Ota K, Kawahara A, Hattori S, Iwama E, Harada T, Matsumoto K, Takayama K, Takamori S, Kage M, Hoshino T (2014). Association of PD-L1 overexpression with activating EGFR mutations in surgically resected nonsmall-cell lung cancer. Ann Oncol.

[52] Zhang Y, Wang L, Li Y, Pan Y, Wang R, Hu H, Li H, Luo X, Ye T, Sun Y, Chen H (2014). Protein expression of programmed death 1 ligand 1 and ligand 2 independently predict poor prognosis in surgically resected lung adenocarcinoma. Onco Targets Ther.

[53] Yang CY, Lin MW, Chang YL, Wu CT, Yang PC (2014). Programmed cell death-ligand 1 expression in surgically resected stage I pulmonary adenocarcinoma and its correlation with driver mutations and clinical outcomes. Eur J Cancer.

[54] Camidge DR, Doebele RC, Kerr KM (2019). Comparing and contrasting predictive biomarkers for immunotherapy and targeted therapy of NSCLC. Nat Rev Clin Oncol.

[55] Joyce JA, Pollard JW (2009). Microenvironmental regulation of metastasis. Nat Rev Cancer.

[56] Quail DF, Joyce JA (2013). Microenvironmental regulation of tumor progression and metastasis. Nat Med.

[57] Schreiber RD, Old LJ, Smyth MJ (2011). Cancer immunoediting:integrating immunity’s roles in cancer suppression and promotion. Science.

[58] DuPage M, Mazumdar C, Schmidt LM, Cheung AF, Jacks T (2012). Expression of tumour-specific antigens underlies cancer immunoediting. Nature..

[59] Dunn GP, Old LJ, Schreiber RD (2004). The immunobiology of cancer immunosurveillance and immunoediting. Immunity.

[60] Balkwill FR, Capasso M, Hagemann T (2012). The tumor microenvironment at a glance. J Cell Sci.

[61] Bruno A, Pagani A, Magnani E, Rossi T, Noonan DM, Cantelmo AR, Albini A (2014). Inflammatory angiogenesis and the tumor microenvironment as targets for cancer therapy and prevention. Advances in Nutrition and Cancer.

[62] Bruno A, Pagani A, Pulze L, Albini A, Dallaglio K, Noonan DM, Mortara L (2014). Orchestration of angiogenesis by immune cells. Front Oncol.

[63] Noonan DM, Barbaro AD, Vannini N, Mortara L, Albini A (2008). Inflammation, inflammatory cells and angiogenesis:decisions and indecisions. Cancer Metastasis Rev.

[64] Bruno A, Ferlazzo G, Albini A, Noonan DM (2014). A think tank of TINK/TANKs:tumor-infiltrating/tumor-associated natural killer cells in tumor progression and angiogenesis. J Natl Cancer Inst.

[65] Mazzaschi G, Madeddu D, Falco A, Bocchialini G, Goldoni M, Sogni F, Armani G, Lagrasta CA, Lorusso B, Mangiaracina C, Vilella R (2018). Low PD-1 expression in cytotoxic CD8+ tumor-infiltrating lymphocytes confers an immune-privileged tissue microenvironment in NSCLC with a prognostic and predictive value. Clin Cancer Res.

[66] Huang SH, Li Y, Zhang J, Rong J, Ye S (2013). Epidermal growth factor receptor-containing exosomes induce tumor-specific regulatory T cells. Cancer Investig.

[67] Zhang B, Zhang Y, Zhao J, Wang Z, Wu T, Ou W, Wang J, Yang B, Zhao Y, Rao Z, Gao J (2014). M2-polarized macrophages contribute to the decreased sensitivity of EGFR-TKIs treatment in patients with advanced lung adenocarcinoma. Med Oncol.

[68] Poggio M, Hu T, Pai CC, Chu B, Belair CD, Chang A, Montabana E, Lang UE, Fu Q, Fong L, Blelloch R (2019). Suppression of Exosomal PD-L1 induces systemic anti-tumor immunity and memory. Cell.

[69] Tosolini M, Kirilovsky A, Mlecnik B, Fredriksen T, Mauger S, Bindea G, Berger A, Bruneval P, Fridman WH, Pagès F, Galon J (2011). Clinical impact of different classes of infiltrating T cytotoxic and helper cells (Th1, th2, treg, th17) in patients with colorectal cancer. Cancer Res.

[70] Fontenot JD, Gavin MA, Rudensky AY (2003). Foxp3 programs the development and function of CD4+ CD25+ regulatory T cells. Nat Immunol.

[71] Frydrychowicz M, Boruczkowski M, Kolecka-Bednarczyk A, Dworacki G (2017). The dual role of Treg in cancer. Scand J Immunol.

[72] Chin AR, Wang SE (2016). Cancer-derived extracellular vesicles:the ‘soil conditioner’in breast cancer metastasis?. Cancer Metastasis Rev.

[73] Whiteside T (2016). Tumor-derived exosomes and their role in tumor-induced immune suppression. Vaccines.

[74] Syn N, Wang L, Sethi G, Thiery JP, Goh BC (2016). Exosome-mediated metastasis:from epithelial–mesenchymal transition to escape from immunosurveillance. Trends Pharmacol Sci.

[75] Fallarino F, Grohmann U, Puccetti P (2012). Indoleamine 2, 3-dioxygenase:from catalyst to signaling function. Eur J Immunol.

[76] Ino K (2011). Indoleamine 2, 3-dioxygenase and immune tolerance in ovarian cancer. Curr Opin Obstet Gynecol.

[77] Chang MH, Ahn HK, Lee J, Jung CK, Choi YL, Park YH, Ahn JS, Park K, Ahn MJ (2011). Clinical impact of amphiregulin expression in patients with epidermal growth factor receptor (EGFR) wild-type nonsmall cell lung cancer treated with EGFR-tyrosine kinase inhibitors. Cancer.

[78] Higginbotham JN, Beckler MD, Gephart JD, Franklin JL, Bogatcheva G, Kremers GJ, Piston DW, Ayers GD, McConnell RE, Tyska MJ, Coffey RJ (2011). Amphiregulin exosomes increase cancer cell invasion. Curr Biol.

[79] Wang S, Zhang Y, Wang Y, Ye P, Li J, Li H, Ding Q, Xia J (2016). Amphiregulin confers regulatory T cell suppressive function and tumor invasion via the EGFR/GSK-3β/Foxp3 axis. J Biol Chem.

[80] Yi T, Lee HL, Cha JH, Ko SI, Kim HJ, Shin HI, Woo KM, Ryoo HM, Kim GS, Baek JH (2008). Epidermal growth factor receptor regulates osteoclast differentiation and survival through cross-talking with RANK signaling. J Cell Physiol.

[81] Salgado R, Denkert C, Demaria S, Sirtaine N, Klauschen F, Pruneri G, Wienert S, Van den Eynden G, Baehner FL, Pénault-Llorca F, Perez EA (2014). The evaluation of tumor-infiltrating lymphocytes (TILs) in breast cancer:recommendations by an international TILs working group 2014. Ann Oncol.

[82] Iglesia Michael D., Parker Joel S., Hoadley Katherine A., Serody Jonathan S., Perou Charles M., Vincent Benjamin G. (2016). Genomic Analysis of Immune Cell Infiltrates Across 11 Tumor Types. Journal of the National Cancer Institute.

[83] Brambilla E, Le Teuff G, Marguet S, Lantuejoul S, Dunant A, Graziano S, Pirker R, Douillard JY, Le Chevalier T, Filipits M, Rosell R (2016). Prognostic effect of tumor lymphocytic infiltration in resectable non–small-cell lung cancer. J Clin Oncol.

[84] Simoni Y, Becht E, Fehlings M, Loh CY, Koo SL, Teng KW, Yeong JP, Nahar R, Zhang T, Kared H, Duan K (2018). Bystander CD8+ T cells are abundant and phenotypically distinct in human tumour infiltrates. Nature.

[85] Teng MW, Ngiow SF, Ribas A, Smyth MJ (2015). Classifying cancers based on T-cell infiltration and PD-L1. Cancer Res.

[86] Haratani K, Hayashi H, Tanaka T, Kaneda H, Togashi Y, Sakai K, Hayashi K, Tomida S, Chiba Y, Yonesaka K, Nonagase Y (2017). Tumor immune microenvironment and nivolumab efficacy in EGFR mutation-positive non-small-cell lung cancer based on T790M status after disease progression during EGFR-TKI treatment. Ann Oncol.

[87] Schalper Kurt Alex, Mani Nikita, Toki Maria, Carvajal-Hausdorf Daniel E., Herbst Roy S., Rimm David L. (2016). Clinical value of measuring T-cell activation and proliferation using multiplexed quantitative fluorescence in non-small cell lung cancer (NSCLC). Journal of Clinical Oncology.

[88] Toki MI, Mani N, Smithy JW, Liu Y, Altan M, Wasserman B, Tuktamyshov R, Schalper K, Syrigos KN, Rimm DL (2018). Immune marker profiling and programmed death ligand 1 expression across NSCLC mutations. J Thorac Oncol.

[89] Yáñez-Mó M, Siljander PR, Andreu Z, Bedina Zavec A, Borràs FE, Buzas EI, Buzas K, Casal E, Cappello F, Carvalho J, Colás E (2015). Biological properties of extracellular vesicles and their physiological functions. J Extracell Vesicles.

[90] Azmi AS, Bao B, Sarkar FH (2013). Exosomes in cancer development, metastasis, and drug resistance:a comprehensive review. Cancer Metastasis Rev.

[91] Zhang C, Ji Q, Yang Y, Li Q, Wang Z (2018). Exosome:function and role in cancer metastasis and drug resistance. Technol Cancer Res Treat.

[92] Steinbichler Teresa Bernadette, Dudás József, Riechelmann Herbert, Skvortsova Ira-Ida (2017). The role of exosomes in cancer metastasis. Seminars in Cancer Biology.

[93] Weidle UH, Birzele F, Kollmorgen G, Rueger R (2017). The multiple roles of exosomes in metastasis. Cancer Genom Proteomics.

[94] Jin H, Wu Y, Tan X (2017). The role of pancreatic cancer-derived exosomes in cancer progress and their potential application as biomarkers. Clin Transl Oncol.

[95] Becker A, Thakur BK, Weiss JM, Kim HS, Peinado H, Lyden D (2016). Extracellular vesicles in cancer:cell-to-cell mediators of metastasis. Cancer Cell.

[96] Melo SA, Sugimoto H, O’Connell JT, Kato N, Villanueva A, Vidal A, Qiu L, Vitkin E, Perelman LT, Melo CA, Lucci A (2014). Cancer exosomes perform cell-independent microRNA biogenesis and promote tumorigenesis. Cancer Cell.

[97] Lobb RJ, Lima LG, Möller A. Exosomes:key mediators of metastasis and pre-metastatic niche formation. In: Seminars in cell & developmental biology. 2017;67:3–10. 10.1016/j.semcdb.2017.01.004 28077297

[98] Allard David, Chrobak Pavel, Allard Bertrand, Messaoudi Nouredin, Stagg John (2019). Targeting the CD73-adenosine axis in immuno-oncology. Immunology Letters.

[99] Streicher Katie, Higgs Brandon W., Wu Song, Coffman Karen, Damera Gautam, Durham Nick, Greenlees Lydia, Lazdun Yelena, Cheng Li, Cooper Zachary, Ranade Koustubh (2017). Increased CD73 and reduced IFNG signature expression in relation to response rates to anti-PD-1(L1) therapies in EGFR-mutant NSCLC. Journal of Clinical Oncology.

[100] Adamiak M, Bujko K, Cymer M, Plonka M, Glaser T, Kucia M, Ratajczak J, Ulrich H, Abdel-Latif A, Ratajczak MZ (2019). Correction:novel evidence that extracellular nucleotides and purinergic signaling induce innate immunity-mediated mobilization of hematopoietic stem/progenitor cells. Leukemia.

[101] Ishii Hidenobu, Azuma Koichi, Kinoshita Takashi, Matsuo Norikazu, Naito Yoshiko, Tokito Takaaki, Yamada Kazuhiko, Hoshino Tomoaki (2018). Predictive value of CD73 expression in EGFR-mutation positive non-small-cell lung cancer patients received immune checkpoint inhibitors. Journal of Clinical Oncology.

[102] Pollack BP, Sapkota B, Cartee TV (2011). Epidermal growth factor receptor inhibition augments the expression of MHC class I and II genes. Clin Cancer Res.

[103] Mortara L, Castellani P, Meazza R, Tosi G, Barbaro AD, Procopio FA, Comes A, Zardi L, Ferrini S, Accolla RS (2006). CIITA-induced MHC class II expression in mammary adenocarcinoma leads to a Th1 polarization of the tumor microenvironment, tumor rejection, and specific antitumor memory. Clin Cancer Res.

[104] Lotem M, Machlenkin A, Hamburger T, Nissan A, Kadouri L, Frankenburg S, Gimmon Z, Elias O, David IB, Kuznetz A, Shiloni E (2009). Autologous melanoma vaccine induces antitumor and self-reactive immune responses that affect patient survival and depend on MHC class II expression on vaccine cells. Clin Cancer Res.

[105] Burns WR, Zhao Y, Frankel TL, Hinrichs CS, Zheng Z, Xu H, Feldman SA, Ferrone S, Rosenberg SA, Morgan RA (2010). A high molecular weight melanoma-associated antigen–specific chimeric antigen receptor redirects lymphocytes to target human melanomas. Cancer Res.

[106] Garrido G, Rabasa A, Garrido C, Chao L, Garrido F, García-Lora ÁM, Sánchez-Ramírez B (2017). Upregulation of HLA class I expression on tumor cells by the anti-EGFR antibody nimotuzumab. Front Pharmacol.

[107] Watanabe S, Hayashi H, Haratani K, Shimizu S, Tanizaki J, Sakai K, Kawakami H, Yonesaka K, Tsurutani J, Togashi Y, Nishio K (2019). Mutational activation of the epidermal growth factor receptor down-regulates major histocompatibility complex class I expression via the extracellular signal-regulated kinase in non–small cell lung cancer. Cancer Sci.

[108] Im JS, Herrmann AC, Bernatchez C, Haymaker C, Molldrem JJ, Hong WK, Perez-Soler R (2016). Immune-modulation by epidermal growth factor receptor inhibitors:implication on anti-tumor immunity in lung cancer. PLoS One.

[109] Kumai T, Matsuda Y, Oikawa K, Aoki N, Kimura S, Harabuchi Y, Celis E, Kobayashi H (2013). EGFR inhibitors augment antitumour helper T-cell responses of HER family-specific immunotherapy. Br J Cancer.

[110] Venugopalan A, Lee MJ, Niu G, Medina-Echeverz J, Tomita Y, Lizak MJ, Cultraro CM, Simpson RM, Chen X, Trepel JB, Guha U (2016). EGFR-targeted therapy results in dramatic early lung tumor regression accompanied by imaging response and immune infiltration in EGFR mutant transgenic mouse models. Oncotarget.

[111] Garrido G, Rabasa A, Garrido C, Lopez A, Chao L, García-Lora ÁM, Garrido F, Fernández LE, Sánchez B (2014). Preclinical modeling of EGFR-specific antibody resistance:oncogenic and immune-associated escape mechanisms. Oncogene.

[112] Herbst RS, Heymach JV, Lippman SM (2008). Lung cancer. N Engl J Med.

[113] Mitsudomi T, Yatabe Y (2010). Epidermal growth factor receptor in relation to tumor development:EGFR gene and cancer. FEBS J.

[114] Wu J.-Y., Yu C.-J., Chang Y.-C., Yang C.-H., Shih J.-Y., Yang P.-C. (2011). Effectiveness of Tyrosine Kinase Inhibitors on "Uncommon" Epidermal Growth Factor Receptor Mutations of Unknown Clinical Significance in Non-Small Cell Lung Cancer. Clinical Cancer Research.

[115] Arcila ME, Nafa K, Chaft JE, Rekhtman N, Lau C, Reva BA, Zakowski MF, Kris MG, Ladanyi M (2013). EGFR exon 20 insertion mutations in lung adenocarcinomas:prevalence, molecular heterogeneity, and clinicopathologic characteristics. Mol Cancer Ther.

[116] Oxnard GR, Lo PC, Nishino M, Dahlberg SE, Lindeman NI, Butaney M, Jackman DM, Johnson BE, Jänne PA (2013). Natural history and molecular characteristics of lung cancers harboring EGFR exon 20 insertions. J Thorac Oncol.

[117] Kobayashi Y, Togashi Y, Yatabe Y, Mizuuchi H, Jangchul P, Kondo C, Shimoji M, Sato K, Suda K, Tomizawa K, Takemoto T (2015). EGFR exon 18 mutations in lung cancer:molecular predictors of augmented sensitivity to afatinib or neratinib as compared with first-or third-generation TKIs. Clin Cancer Res.

[118] Yamada T, Hirai S, Katayama Y, Yoshimura A, Shiotsu S, Watanabe S, Kikuchi T, Hirose K, Kubota Y, Chihara Y, Harada T (2019). Retrospective efficacy analysis of immune checkpoint inhibitors in patients with EGFR-mutated non-small cell lung cancer. Cancer medicine.

[119] Yoshida H, Kim YH, Ozasa H, Nagai H, Sakamori Y, Tsuji T, Nomizo T, Yasuda Y, Funazo T, Hirai T (2017). Nivolumab in non-small-cell lung cancer with EGFR mutation. Ann Oncol.

[120] Gettinger S, Horn L, Jackman D, Spigel D, Antonia S, Hellmann M, Powderly J, Heist R, Sequist LV, Smith DC, Leming P (2018). Five-year follow-up of nivolumab in previously treated advanced non–small-cell lung cancer:results from the CA209-003 study. J Clin Oncol.

[121] Abdelhamed S, Ogura K, Yokoyama S, Saiki I, Hayakawa Y (2016). AKT-STAT3 pathway as a downstream target of EGFR signaling to regulate PD-L1 expression on NSCLC cells. J Cancer.

[122] D'incecco A, Andreozzi M, Ludovini V, Rossi E, Capodanno A, Landi L, Tibaldi C, Minuti G, Salvini J, Coppi E, Chella A (2015). PD-1 and PD-L1 expression in molecularly selected non-small-cell lung cancer patients. Br J Cancer.

[123] Lin PL, Wu TC, Wu DW, Wang L, Chen CY, Lee H (2017). An increase in BAG-1 by PD-L1 confers resistance to tyrosine kinase inhibitor in non–small cell lung cancer via persistent activation of ERK signalling. Eur J Cancer.

[124] Lin K, Cheng J, Yang T, Li Y, Zhu B (2015). EGFR-TKI down-regulates PD-L1 in EGFR mutant NSCLC through inhibiting NF-κB. Biochem Biophys Res Commun.

[125] Zhang W, Pang Q, Yan C, Wang Q, Yang J, Yu S, Liu X, Yuan Z, Wang P, Xiao Z (2017). induction of PD-l1 expression by epidermal growth factor receptor–mediated signaling in esophageal squamous cell carcinoma. Onco Targets Ther.

[126] Ota K, Azuma K, Kawahara A, Hattori S, Iwama E, Tanizaki J, Harada T, Matsumoto K, Takayama K, Takamori S, Kage M (2015). Induction of PD-L1 expression by the EML4–ALK oncoprotein and downstream signaling pathways in non–small cell lung cancer. Clin Cancer Res.

[127] Chen N, Fang W, Zhan J, Hong S, Tang Y, Kang S, Zhang Y, He X, Zhou T, Qin T, Huang Y (2015). Upregulation of PD-L1 by EGFR activation mediates the immune escape in EGFR-driven NSCLC:implication for optional immune targeted therapy for NSCLC patients with EGFR mutation. J Thorac Oncol.

[128] Lastwika K, Wilson W, Dennis PA. PI3K/AKT/mTOR pathway activation drives expression of the immune inhibitory ligand PD-L1 in NSCLC. Cancer Res. 73:4981–4981. 10.1158/1538-7445.AM2013-4981.

[129] Yokogami K, Wakisaka S, Avruch J, Reeves SA (2000). Serine phosphorylation and maximal activation of STAT3 during CNTF signaling is mediated by the rapamycin target mTOR. Curr Biol.

[130] Okita R, Maeda A, Shimizu K, Nojima Y, Saisho S, Nakata M (2017). PD-L1 overexpression is partially regulated by EGFR/HER2 signaling and associated with poor prognosis in patients with non-small-cell lung cancer. Cancer Immunol Immunother.

[131] Zhang N, Zeng Y, Du W, Zhu J, Shen D, Liu Z, Huang JA (2016). The EGFR pathway is involved in the regulation of PD-L1 expression via the IL-6/JAK/STAT3 signaling pathway in EGFR-mutated non-small cell lung cancer. Int J Oncol.

[132] Cheng CC, Lin HC, Tsai KJ, Chiang YW, Lim KH, Chen CG, Su YW, Peng CL, Ho AS, Huang L, Chang YC (2018). Epidermal growth factor induces STAT1 expression to exacerbate the IFNr-mediated PD-L1 axis in epidermal growth factor receptor-positive cancers. Mol Carcinog.

[133] Gao SP, Mark KG, Leslie K, Pao W, Motoi N, Gerald WL, Travis WD, Bornmann W, Veach D, Clarkson B, Bromberg JF (2007). Mutations in the EGFR kinase domain mediate STAT3 activation via IL-6 production in human lung adenocarcinomas. J Clin Invest.

[134] Li CW, Lim SO, Xia W, Lee HH, Chan LC, Kuo CW, Khoo KH, Chang SS, Cha JH, Kim T, Hsu JL (2016). Glycosylation and stabilization of programmed death ligand-1 suppresses T-cell activity. Nat Commun.

[135] Takada K, Toyokawa G, Tagawa T, Kohashi K, Shimokawa M, Akamine T, Takamori S, Hirai F, Shoji F, Okamoto T, Oda Y, Maehara Y (2018). PD-L1 expression according to the EGFR status in primary lung adenocarcinoma. Lung Cancer.

[136] Lee J, Park CK, Yoon HK, Sa YJ, Woo IS, Kim HR, Kim SY, Kim TJ (2019). PD-L1 expression in ROS1-rearranged non-small cell lung cancer:a study using simultaneous genotypic screening of EGFR, ALK, and ROS1. Thorac Cancer.

[137] Heigener DF, Reck M (2018). Impact of PD-L1 expression in EGFR-positive NSCLC? The answer remains the same. J Thorac Oncol.

[138] Ji M, Liu Y, Li Q, Li X, Ning Z, Zhao W, Shi H, Jiang J, Wu C (2016). PD-1/PD-L1 expression in non-small-cell lung cancer and its correlation with EGFR/KRAS mutations. Cancer Biology Ther.

[139] Li J, Chen Y, Shi X, Le X, Feng F, Chen J, Zhou C, Chen Y, Wen S, Zeng H, Chen AM (2017). A systematic and genome-wide correlation meta-analysis of PD-L1 expression and targetable NSCLC driver genes. J Thorac Dis.

[140] Hersom M, Jørgensen JT (2018). Companion and complementary diagnostics–focus on PD-L1 expression assays for PD-1/PD-L1 checkpoint inhibitors in non–small cell lung Cancer. Ther Drug Monit.

[141] Büttner R, Gosney JR, Skov BG, Adam J, Motoi N, Bloom KJ, Dietel M, Longshore JW, López-Ríos F, Penault-Llorca F, Viale G (2017). Programmed death-ligand 1 immunohistochemistry testing:a review of analytical assays and clinical implementation in non–small-cell lung cancer. J Clin Oncol.

[142] Nakamura S, Hayashi K, Imaoka Y, Kitamura Y, Akazawa Y, Tabata K, Groen R, Tsuchiya T, Yamasaki N, Nagayasu T, Fukuoka J (2017). Intratumoral heterogeneity of programmed cell death ligand-1 expression is common in lung cancer. PLoS One.

[143] Taube JM (2014). Unleashing the immune system:PD-1 and PD-ls in the pre-treatment tumor microenvironment and correlation with response to PD-1/PD-L1 blockade. Oncoimmunology.

[144] Berchuck A, Olt GJ, Soisson AP, Kamel A, Soper JT, Boyer CM, Clarke-Pearson DL, Leslie DS, Bast RC (1990). Heterogeneity of antigen expression in advanced epithelial ovarian cancer. Am J Obstet Gynecol.

[145] Passiglia F, Bronte G, Bazan V, Natoli C, Rizzo S, Galvano A, Listì A, Cicero G, Rolfo C, Santini D, Russo A (2016). PD-L1 expression as predictive biomarker in patients with NSCLC:a pooled analysis. Oncotarget.

[146] Noguchi T, Ward JP, Gubin MM, Arthur CD, Lee SH, Hundal J, Selby MJ, Graziano RF, Mardis ER, Korman AJ, Schreiber RD (2017). Temporally distinct PD-L1 expression by tumor and host cells contributes to immune escape. Cancer Immunol Res.

[147] Cohen MH, Williams GA, Sridhara R, Chen G, McGuinn WD, Morse D, Abraham S, Rahman A, Liang C, Lostritto R, Baird A (2004). United States Food and Drug Administration drug approval summary:gefitinib (ZD1839; Iressa) tablets. Clin Cancer Res.

[148] Cohen MH, Johnson JR, Chen YF, Sridhara R, Pazdur R (2005). FDA drug approval summary:erlotinib (Tarceva®) tablets. Oncologist.

[149] Miller VA, Hirsh V, Cadranel J, Chen YM, Park K, Kim SW, Zhou C, Su WC, Wang M, Sun Y, Heo DS (2012). Afatinib versus placebo for patients with advanced, metastatic non-small-cell lung cancer after failure of erlotinib, gefitinib, or both, and one or two lines of chemotherapy (LUX-lung 1):a phase 2b/3 randomised trial. Lancet Oncol.

[150] Park K, Tan EH, O’Byrne K, Zhang L, Boyer M, Mok T, Hirsh V, Yang JC, Lee KH, Lu S, Shi Y (2016). Afatinib versus gefitinib as first-line treatment of patients with EGFR mutation-positive non-small-cell lung cancer (LUX-lung 7):a phase 2B, open-label, randomised controlled trial. Lancet Oncol.

[151] Suda K, Rivard CJ, Mitsudomi T, Hirsch FR (2017). Overcoming resistance to EGFR tyrosine kinase inhibitors in lung cancer, focusing on non-T790M mechanisms. Expert Rev Anticancer Ther.

[152] Hata A, Katakami N, Nanjo S, Okuda C, Kaji R, Masago K, Fujita S, Yoshida H, Zama K, Imai Y, Hirata Y (2017). Programmed death-ligand 1 expression and T790M status in EGFR-mutant non-small cell lung cancer. Lung Cancer.

[153] Dominguez C, Tsang KY, Palena C (2016). Short-term EGFR blockade enhances immune-mediated cytotoxicity of EGFR mutant lung cancer cells:rationale for combination therapies. Cell Death Dis.

[154] Brea EJ, Oh CY, Manchado E, Budhu S, Gejman RS, Mo G, Mondello P, Han JE, Jarvis CA, Ulmert D, Xiang Q (2016). Kinase regulation of human MHC class I molecule expression on cancer cells. Cancer Immunol Res.

[155] Helland Å, Brustugun OT, Nakken S, Halvorsen AR, Dønnem T, Bremnes R, Busund LT, Sun J, Lorenz S, Solberg SK, Jørgensen LH (2017). High number of kinome-mutations in non-small cell lung cancer is associated with reduced immune response and poor relapse-free survival. Int J Cancer.

[156] Busch SE, Hanke ML, Kargl J, Metz HE, MacPherson D, Houghton AM (2016). Lung cancer subtypes generate unique immune responses. J Immunol.

[157] Sawanobori Y, Ueha S, Kurachi M, Shimaoka T, Talmadge JE, Abe J, Shono Y, Kitabatake M, Kakimi K, Mukaida N, Matsushima K (2008). Chemokine-mediated rapid turnover of myeloid-derived suppressor cells in tumor-bearing mice. Blood.

[158] Chang AL, Miska J, Wainwright DA, Dey M, Rivetta CV, Yu D, Kanojia D, Pituch KC, Qiao J, Pytel P, Han Y (2016). CCL2 produced by the glioma microenvironment is essential for the recruitment of regulatory T cells and myeloid-derived suppressor cells. Cancer Res.

[159] Yamaki M, Sugiura K, Muro Y, Shimoyama Y, Tomita Y (2010). Epidermal growth factor receptor tyrosine kinase inhibitors induce CCL2 and CCL5 via reduction in IL-1R2 in keratinocytes. Exp Dermatol.

[160] Paul T, Schumann C, Rüdiger S, Boeck S, Heinemann V, Kächele V, Steffens M, Scholl C, Hichert V, Seufferlein T, Stingl JC (2014). Cytokine regulation by epidermal growth factor receptor inhibitors and epidermal growth factor receptor inhibitor associated skin toxicity in cancer patients. Eur J Cancer.

[161] Gabrilovich DI, Nagaraj S (2009). Myeloid-derived suppressor cells as regulators of the immune system. Nat Rev Immunol.

[162] Millrud CR, Bergenfelz C, Leandersson K. On the origin of myeloid- derived suppressor cells. Oncotarget. 2017:3649–65. 10.18632/oncotarget.12278 PMC5356220 27690299

[163] Emmanuel C, Gava N, Kennedy C, Balleine RL, Sharma R, Wain G, Brand A, Hogg R, Etemadmoghadam D, George J, Birrer MJ (2011). Comparison of expression profiles in ovarian epithelium in vivo and ovarian cancer identifies novel candidate genes involved in disease pathogenesis. PLoS One.

[164] Andresen E, Günther G, Bullwinkel J, Lange C, Heine H (2011). Increased expression of beta-defensin 1 (DEFB1) in chronic obstructive pulmonary disease. PLoS One.

[165] De Santa F, Narang V, Yap ZH, Tusi BK, Burgold T, Austenaa L, Bucci G, Caganova M, Notarbartolo S, Casola S, Testa G (2009). Jmjd3 contributes to the control of gene expression in LPS-activated macrophages. EMBO J.

[166] Cao R, Wang L, Wang H, Xia L, Erdjument-Bromage H, Tempst P, Jones RS, Zhang Y (2002). Role of histone H3 lysine 27 methylation in Polycomb-group silencing. Science..

[167] Boyer LA, Plath K, Zeitlinger J, Brambrink T, Medeiros LA, Lee TI, Levine SS, Wernig M, Tajonar A, Ray MK, Bell GW (2006). Polycomb complexes repress developmental regulators in murine embryonic stem cells. Nature.

[168] Hahn MA, Hahn T, Lee DH, Esworthy RS, Kim BW, Riggs AD, Chu FF, Pfeifer GP (2008). Methylation of polycomb target genes in intestinal cancer is mediated by inflammation. Cancer Res.

[169] Ishii M, Wen H, Corsa CA, Liu T, Coelho AL, Allen RM, Carson WF, Cavassani KA, Li X, Lukacs NW, Hogaboam CM (2009). Epigenetic regulation of the alternatively activated macrophage phenotype. Blood.

[170] Chen X, El Gazzar M, Yoza BK, McCall CE (2009). The NF-κB factor RelB and histone H3 lysine methyltransferase G9a directly interact to generate epigenetic silencing in endotoxin tolerance. J Biol Chem.

[171] Zhou J, Qu Z, Sun F, Han L, Li L, Yan S, Stabile LP, Chen LF, Siegfried JM, Xiao G (2017). Myeloid STAT3 promotes lung tumorigenesis by transforming tumor immunosurveillance into tumor-promoting inflammation. Cancer Immunol Res.

[172] El Gazzar M, Yoza BK, Chen X, Hu J, Hawkins GA, McCall CE (2008). G9a and HP1 couple histone and DNA methylation to TNFα transcription silencing during endotoxin tolerance. J Biol Chem.

[173] Bird AP (1986). CpG-rich islands and the function of DNA methylation. Nature.

[174] Hermann A, Gowher H, Jeltsch A (2004). Biochemistry and biology of mammalian DNA methyltransferases. Cell Mol Life Sci.

[175] Wu AA, Drake V, Huang HS, Chiu S, Zheng L (2015). Reprogramming the tumor microenvironment:tumor-induced immunosuppressive factors paralyze T cells. Oncoimmunology.

[176] Han JJ, Kim DW, Koh J, Keam B, Kim TM, Jeon YK, Lee SH, Chung DH, Heo DS (2016). Change in PD-L1 expression after acquiring resistance to gefitinib in EGFR-mutant non–small-cell lung cancer. Clin Lung Cancer.

[177] Hsu KH, Huang YH, Tseng JS, Chen KC, Ku WH, Su KY, Chen JJ, Chen HW, Yu SL, Yang TY, Chang GC (2019). High PD-L1 expression correlates with primary resistance to EGFR-TKIs in treatment naïve advanced EGFR-mutant lung adenocarcinoma patients. Lung Cancer.

[178] Su S, Dong ZY, Xie Z, Yan LX, Li YF, Su J, Liu SY, Yin K, Chen RL, Huang SM, Chen ZH (2018). Strong programmed death ligand 1 expression predicts poor response and De novo resistance to EGFR tyrosine kinase inhibitors among NSCLC patients with EGFR mutation. J Thorac Oncol.

[179] Soria JC, Wu YL, Nakagawa K, Kim SW, Yang JJ, Ahn MJ, Wang J, Yang JC, Lu Y, Atagi S, Ponce S (2015). Gefitinib plus chemotherapy versus placebo plus chemotherapy in EGFR-mutation-positive non-small-cell lung cancer after progression on first-line gefitinib (IMPRESS): a phase 3 randomised trial. Lancet Oncol.

[180] Lisberg A, Cummings A, Goldman JW, Bornazyan K, Reese N, Wang T, Coluzzi P, Ledezma B, Mendenhall M, Hunt J, Wolf B (2018). A phase II study of pembrolizumab in EGFR-mutant, PD-L1+, tyrosine kinase inhibitor naïve patients with advanced NSCLC. J Thorac Oncol.

[181] Hegde PS, Wallin JJ, Mancao C (2018). Predictive markers of anti-VEGF and emerging role of angiogenesis inhibitors as immunotherapeutics. Semin Cancer Biol.

[182] Gabrilovich DI, Chen HL, Girgis KR, Cunningham HT, Meny GM, Nadaf S, Kavanaugh D, Carbone DP (1996). Production of vascular endothelial growth factor by human tumors inhibits the functional maturation of dendritic cells. Nat Med.

[183] Motz GT, Santoro SP, Wang LP, Garrabrant T, Lastra RR, Hagemann IS, Lal P, Feldman MD, Benencia F, Coukos G (2014). Tumor endothelium FasL establishes a selective immune barrier promoting tolerance in tumors. Nat Med.

[184] Hodi FS, Lawrence D, Lezcano C, Wu X, Zhou J, Sasada T, Zeng W, Giobbie-Hurder A, Atkins MB, Ibrahim N, Friedlander P (2014). Bevacizumab plus ipilimumab in patients with metastatic melanoma. Cancer Immunol Res.

[185] Wallin JJ, Bendell JC, Funke R, Sznol M, Korski K, Jones S, Hernandez G, Mier J, He X, Hodi FS, Denker M (2016). Atezolizumab in combination with bevacizumab enhances antigen-specific T-cell migration in metastatic renal cell carcinoma. Nat Commun.

[186] Reck M, Mok TS, Nishio M, Jotte RM, Cappuzzo F, Orlandi F, Stroyakovskiy D, Nogami N, Rodríguez-Abreu D, Moro-Sibilot D, Thomas CA (2019). Atezolizumab plus bevacizumab and chemotherapy in non-small-cell lung cancer (IMpower150): key subgroup analyses of patients with EGFR mutations or baseline liver metastases in a randomised, open-label phase 3 trial. Lancet Respir Med.

[187] Blakely CM, Watkins TB, Wu W, Gini B, Chabon JJ, McCoach CE, McGranahan N, Wilson GA, Birkbak NJ, Olivas VR, Rotow J (2017). Evolution and clinical impact of co-occurring genetic alterations in advanced-stage EGFR-mutant lung cancers. Nat Genet.

[188] Gibbons DL, Chow LQ, Kim DW, Kim SW, Yeh T, Song X, Jiang H, Taylor R, Karakunnel J, Creelan B (2016). 57O efficacy, safety and tolerability of MEDI4736 (durvalumab [D]), a human IgG1 anti-programmed cell death-ligand-1 (PD-L1) antibody, combined with gefitinib (G): a phase I expansion in TKI-naïve patients (pts) with EGFR mutant NSCLC. J Thorac oncol.

[189] Planchard D, Barlesi F, Gomez-Roca C, Mazieres J, Varga A, Greillier L, Chaput N, Parlavecchio C, Malekzadeh K, Ngocamus M, Zahi S. Phase I, safety, tolerability and preliminary efficacy study of tremelimumab (Trem) in combination with gefitinib (Gef) in EGFR-mutant (EGFR-mut) NSCLC (GEFTREM). Ann Oncol. 2016;27(6):416–454. 10.1093/annonc/mdw383.

[190] Spigel DR, Reynolds C, Waterhouse D, Garon EB, Chandler J, Babu S, Thurmes P, Spira A, Jotte R, Zhu J, Lin WH (2018). Phase 1/2 study of the safety and tolerability of nivolumab plus crizotinib for the first-line treatment of anaplastic lymphoma kinase translocation—positive advanced non–small cell lung cancer (CheckMate 370). J Thorac Oncol.

[191] Gettinger S, Rizvi N, Chow LQ, Borghaei H, Brahmer JR, Juergens R, Shepherd FA, Laurie SA, Gerber DE, Goldman J, Shen Y (2014). 1054PD Nivolumab (anti-PD-1; BMS-936558, ONO-4538) in combination with platinum-based doublet chemotherapy (PT-DC) or erlotinib (ERL) in advanced non-small cell lung cancer (NSCLC). Ann Oncol.

[192] Ma BB, Rudin CM, Cervantes A, Dowlati A, Costa D, Schmid P, Heist R, Villaflor VM, Sarkar I, Huseni MA, Foster P. 441O Preliminary safety and clinical activity of erlotinib plus atezolizumab from a Phase Ib study in advanced NSCLC. Ann Oncol. 2016;27(9). 10.1093/annonc/mdw594.005

[193] Ahn MJ, Yang J, Yu H, Saka H, Ramalingam S, Goto K (2016). Osimertinib combined with durvalumab in EGFR-mutant non-small cell lung cancer: results from the TATTON phase Ib trial. J Thorac Oncol.

[194] Garassino MC, Cho BC, Kim JH, Mazieres J, Gray JE, Wheatley-Price P, Park K, Soo RA, Huang Y, Wadsworth C, Dennis PA. Durvalumab in≥ 3rd-line advanced NSCLC: Updated results from the phase 2 ATLANTIC study. Lancet Oncol. 2018;19:521–536.

[195] Cappuzzo F, McCleod M, Hussein M, Morabito A, Rittmeyer A, Conter HJ, Kopp HG, Daniel D, Mccune S, Mekhail T, Zer A (2018). LBA53 IMpower130: progression-free survival (PFS) and safety analysis from a randomised Phase III study of carboplatin+ nab-paclitaxel (CnP) with or without atezolizumab (atezo) as first-line (1L) therapy in advanced non-squamous NSCLC. Ann Oncol.

